# Supplementary material for: Topography and human pressure in mountain ranges alter expected species responses to climate change
Source: Nat Commun. 2020 Apr 24;11:1974. doi: 10.1038/s41467-020-15881-x (PMC7181879; doi:10.1038/s41467-020-15881-x)
Supplement: Supplementary file 1 — Supplementary Information [file 41467_2020_15881_MOESM1_ESM.pdf]

Topography and human pressure in mountain ranges alter expected species responses to climate change

Paul R. Elsen[1,2]*, William B. Monahan[3], Adina M. Merenlender[1]

[1]Department of Environmental Science, Policy, and Management, University of California, Berkeley, Berkeley, CA, 94720 USA
[2]Wildlife Conservation Society, 2300 Southern Boulevard, Bronx, NY, 10460 USA
[3]USDA Forest Service, Forest Health Protection, Fort Collins, CO, 80526 USA

*e-mail: pelsen@wcs.org

Supplementary Figures

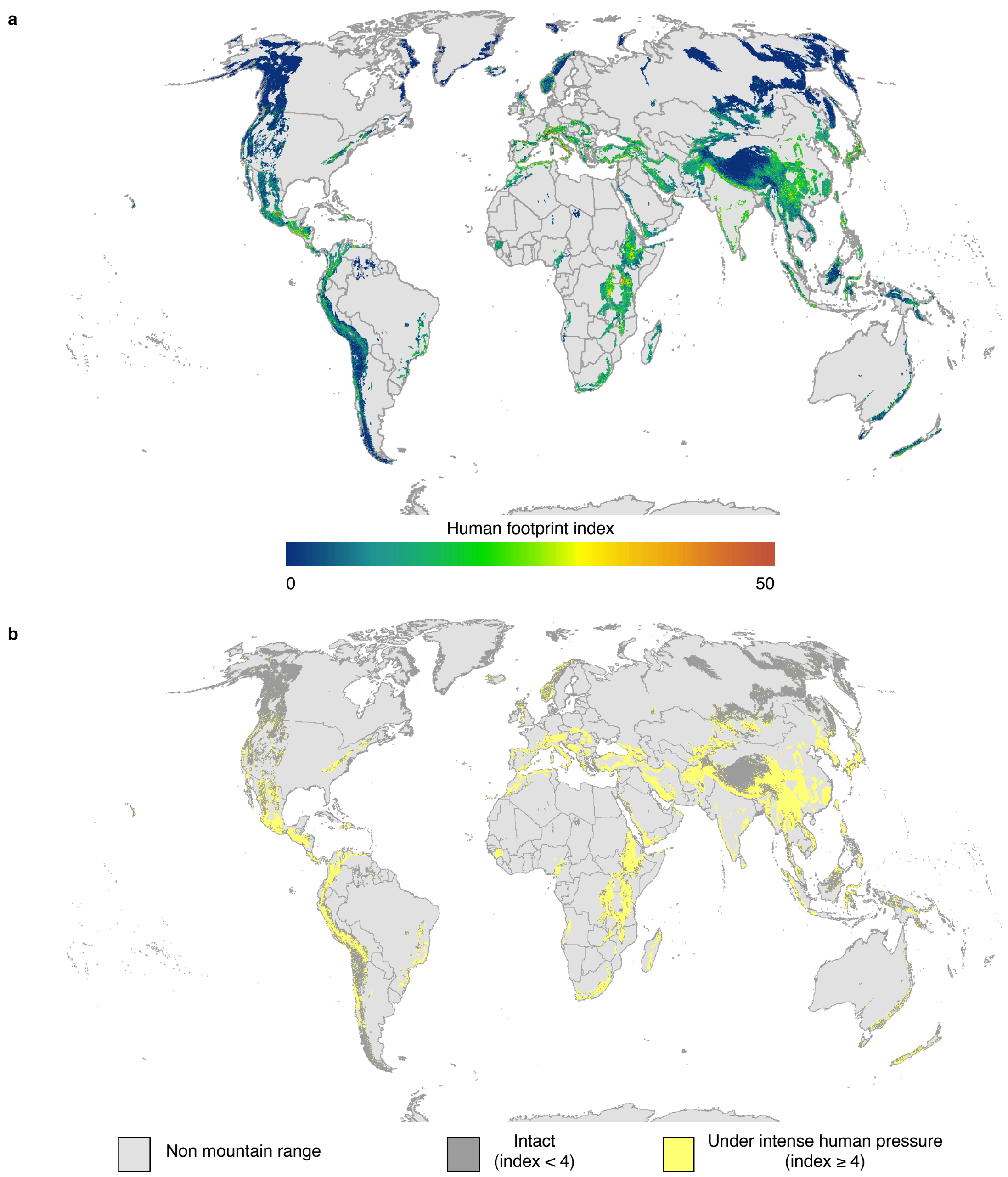

Supplementary Figure 1. Human pressure in global mountain ranges. Map of the Human Footprint index (HFI) within global mountain ranges ranging from 0 (no human pressure) to 50 (maximum human pressure) (**a**) and the same HFI with all values < 4 considered intact (mapped in grey) and all values ≥ 4 in considered under intense human pressure (mapped in yellow; **b**).

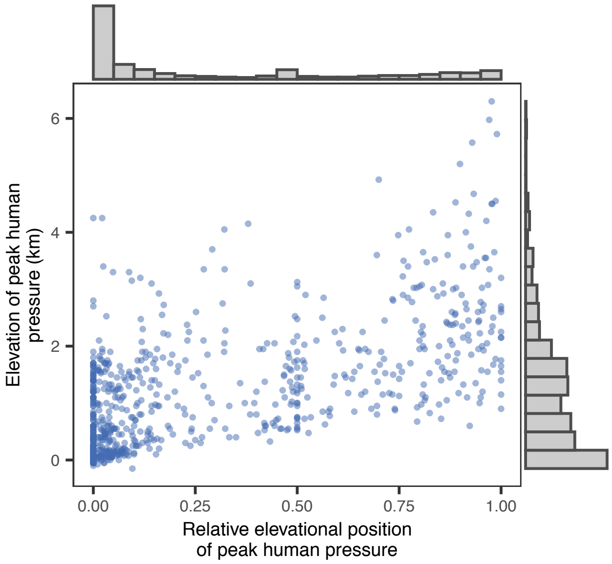

Supplementary Figure 2. Human pressure along elevational gradients for global mountain ranges. Elevation (*y*-axis) and relative elevational position (*x*-axis) of peak human pressure within global mountain ranges (blue points, *n* = 1,010). Relative elevational position refers to the position along the elevational gradient where human pressure is maximal (e.g., a value of 0.5 equates to peak human pressure halfway up the elevational gradient from a mountain range's base). Marginal histograms show distributions for the variable on the opposing axis.

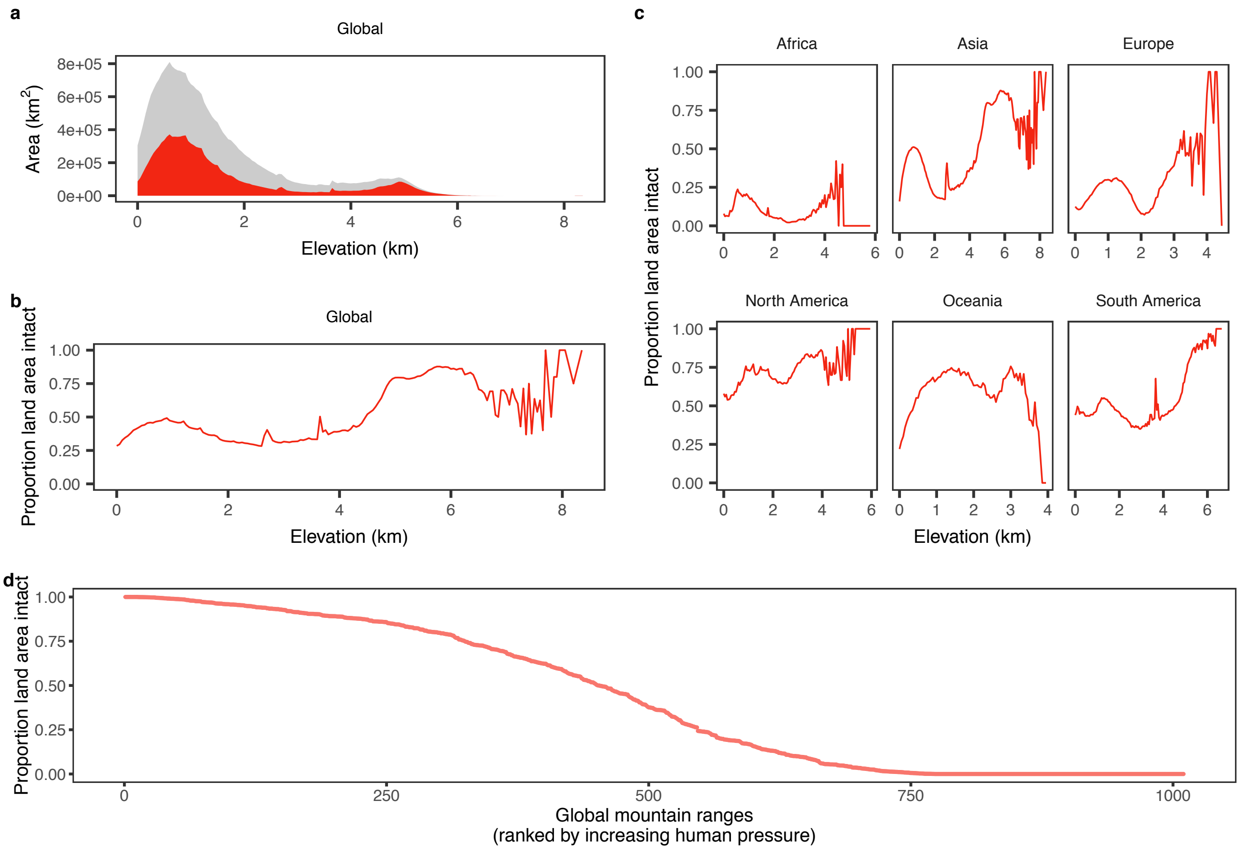

Supplementary Figure 3. Global and regional trends in human pressure over elevational gradients in mountain ranges. The global amount (**a**) and proportion (**b**) of intact land area over elevation. (**c**) as in (**b**) at the continental scale. (**d**) The distribution of 1,010 mountain ranges considered in this study, ranked by proportion of intact land area.

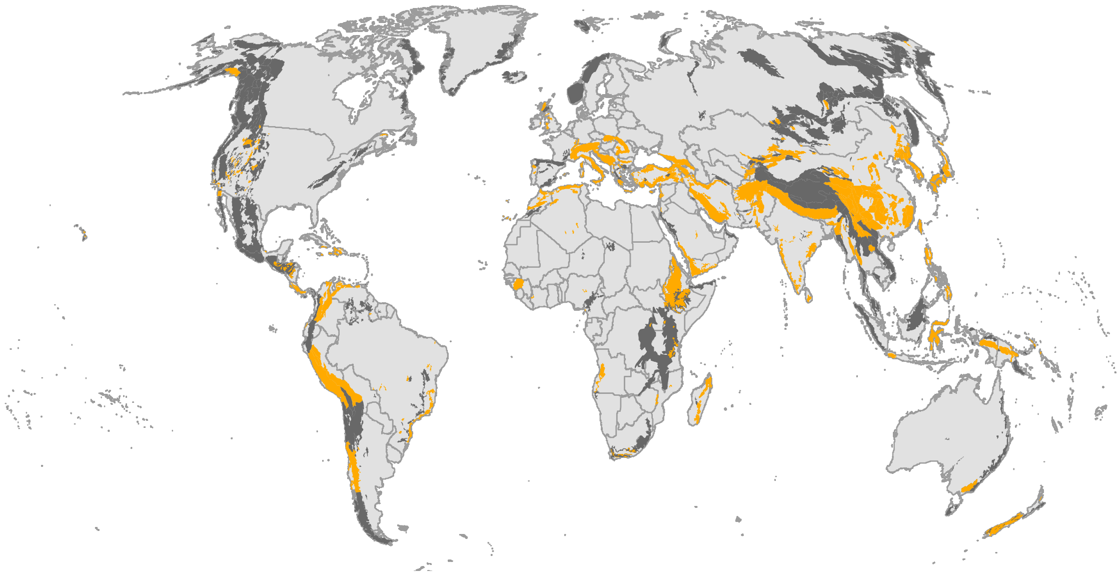

Supplementary Figure 4. Mountain topography reclassifications accounting for human pressure. Map of global mountain ranges highlighting those that changed mountain classifications (orange) when considering total versus intact land area elevational distributions (see Fig. 1 for map comparisons of mountain classifications).

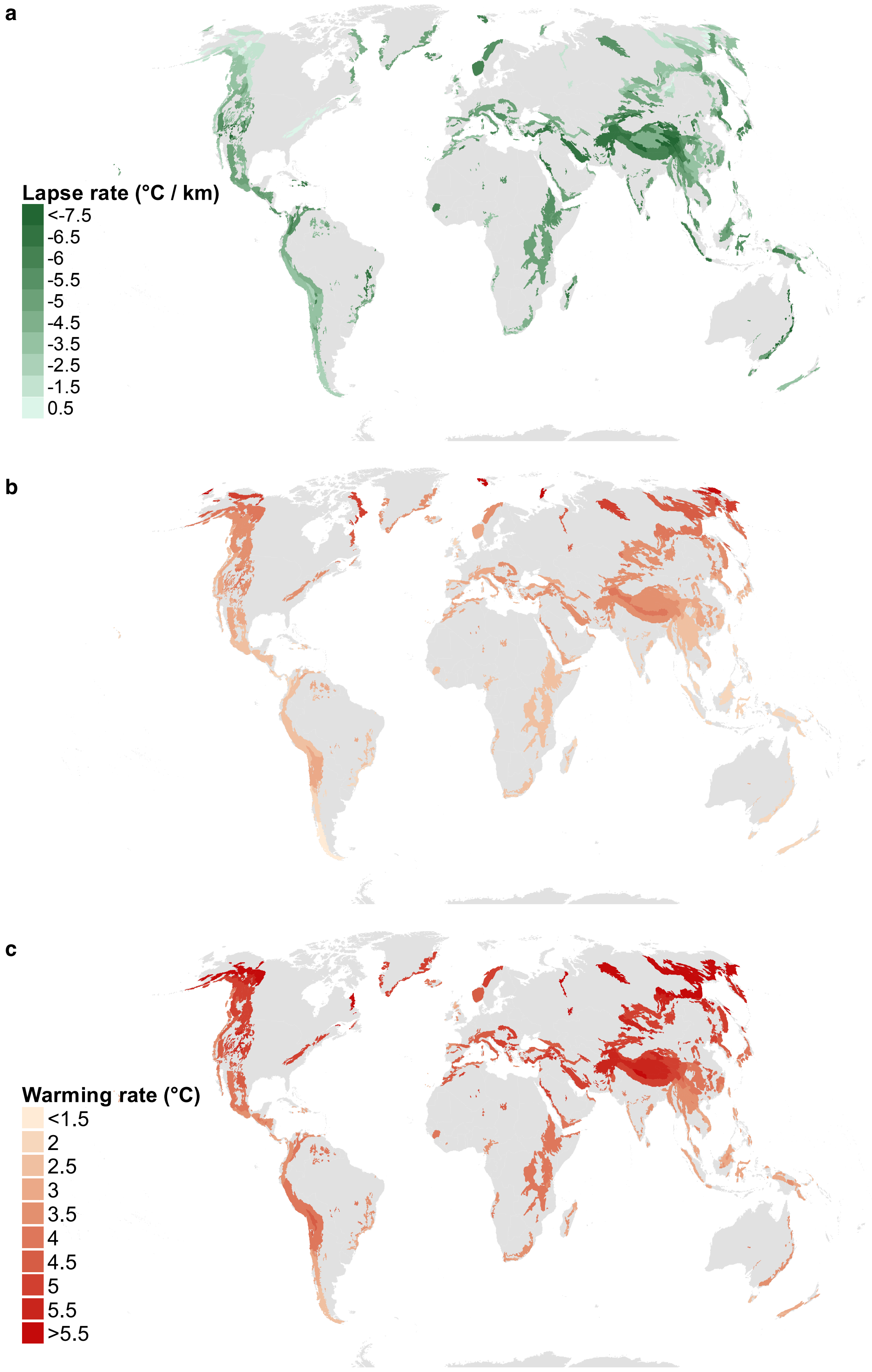
a
Lapse rate (°C / km)
<-7.5
-6.5
-6
-5.5
-5
-4.5
-3.5
-2.5
-1.5
0.5
b
c
Warming rate (°C)
<1.5
2
2.5
3
3.5
4
4.5
5
5.5
>5.5

Supplementary Figure 5. Adiabatic lapse and warming rates for global mountain ranges. Maps of adiabatic lapse rates (**a**) and average warming rates across 17 GCMs using RCP 4.5 (**b**) and RCP 8.5 (**c**) calculated for the world's mountain ranges (n = 1,010). See Methods for details of lapse and warming rate calculations. See also Supplementary Data File 2 for detailed lapse rate regression result figures for all mountain ranges.

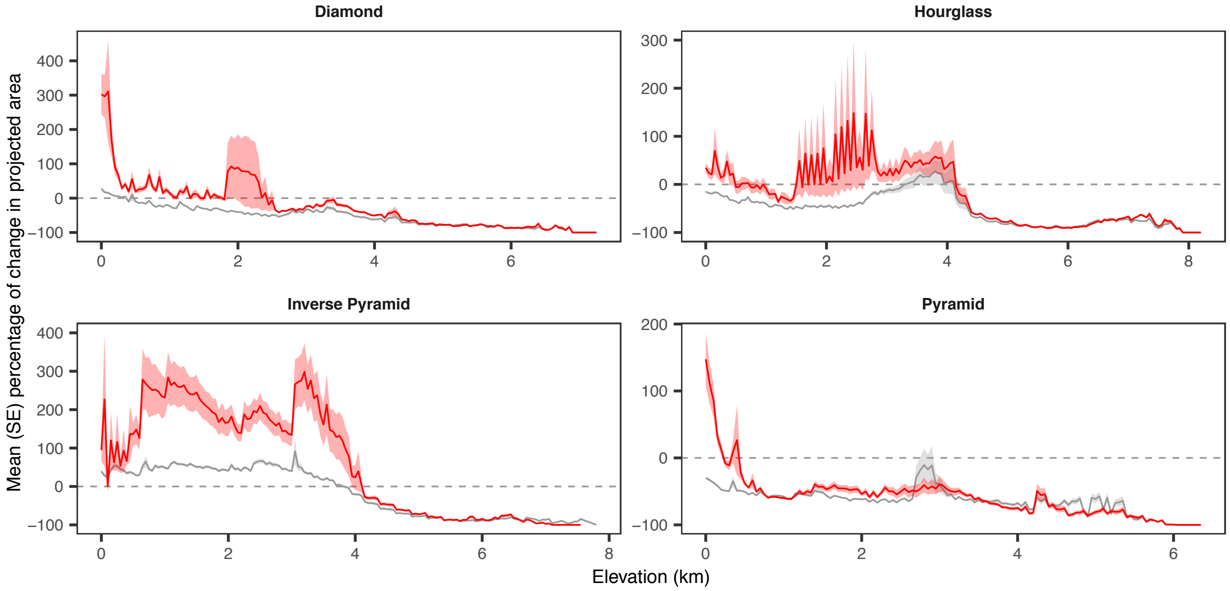

Supplementary Figure 6. Average projected area changes for species undergoing elevational range shifts under RCP 4.5. Mean (lines) and standard error (shaded regions) percentage of change in projected total (grey lines) and intact (red lines) land area across all modeled species and all mountain ranges by mountain classification using RCP 4.5. See Fig. 3a for analogous results using RCP 8.5.

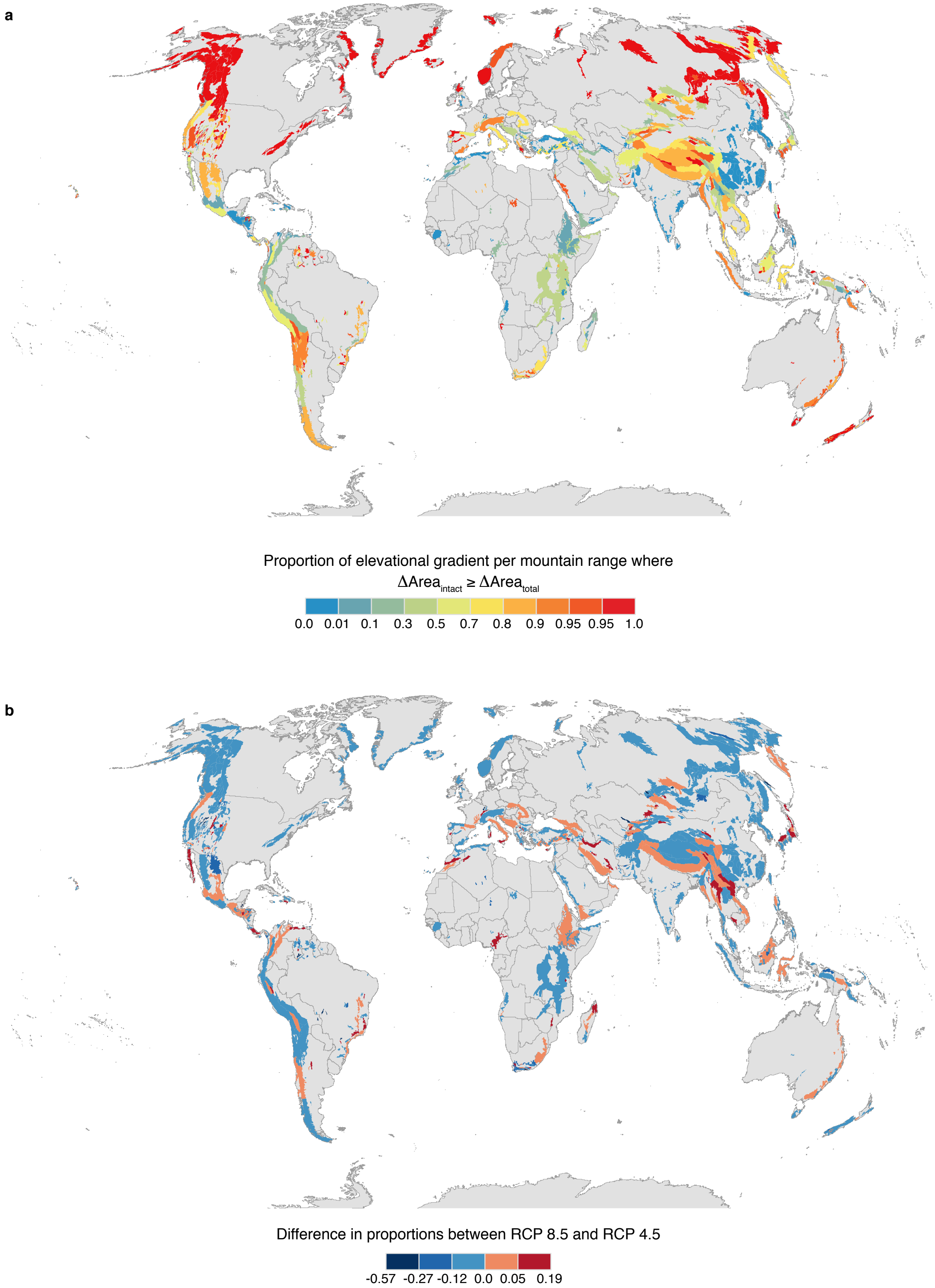
a
Proportion of elevational gradient per mountain range where
ΔArea$_{intact}$ ≥ ΔArea$_{total}$
0.0 0.01 0.1 0.3 0.5 0.7 0.8 0.9 0.95 0.95 1.0
b
Difference in proportions between RCP 8.5 and RCP 4.5
-0.57 -0.27 -0.12 0.0 0.05 0.19

Supplementary Figure 7. Global summary of comparisons of projected area from modeled elevational range shifts with and without accounting for human pressure (**a**) Map of global mountain ranges showing the proportion of the elevational gradient for each range where percentage of change in intact area equals or exceeds the percentage of change in total area following range shifts for a suite of modeled hypothetical montane species. Range shifts are calculated using mountain range-specific rates of mean annual temperature change averaged across 17 GCMs for RCP 4.5 in 2070, mountain range-specific adiabatic lapse rates, and varying elevational range sizes for hypothetical species. See Methods and Supplementary Figure 9 for descriptive and schematic overviews of the modeling procedure, and see Fig. 2 for the analogous global map using RCP 8.5. (**b**) Map of the difference in proportions between results using RCP 8.5 and RCP 4.5.

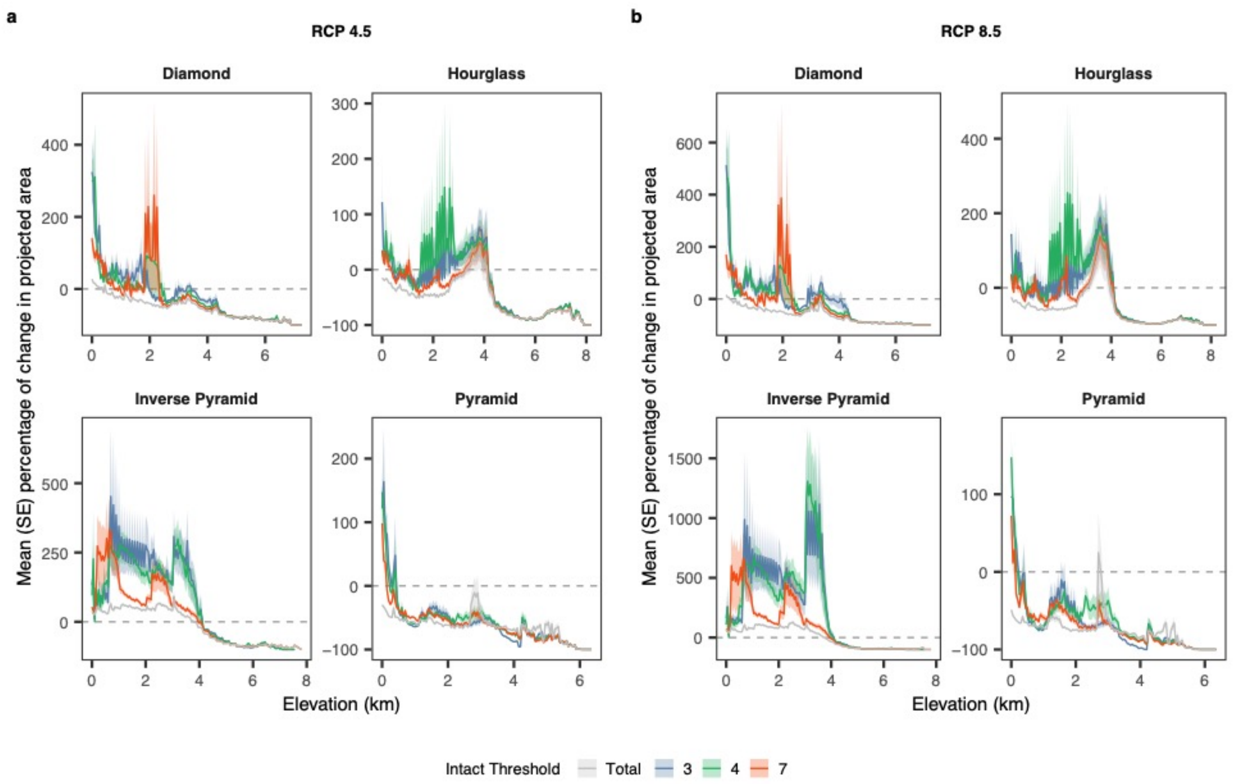

Supplementary Figure 8. Average projected area changes for species undergoing elevational range shifts under different intact thresholds. Mean (lines) and standard error (shaded regions) percentage of change in projected total and intact land area using three different Human Footprint index threshold values by mountain classification under RCP 4.5 (**a**) and RCP 8.5 (**b**). See text and Methods for details of modeled range shifts.

For mountain range *i*

Step 1: Establish number of species, *s*

$s_i = b_i \times (a_i / 100) / 2$ (for even number of bands) or $(b_i - 1) \times ((a_i + 50) / 100)) / 2$ (for odd number of bands)

b_i = number of 50-m elevational bands

a_i = mountain range amplitude, in meters

Step 2: Calculate species' elevational range sizes

Range sizes_i = 100 m, 200 m, ..., a_i m

Step 3: Distribute species over elevational gradient

Set species' lower range limit every 50 m starting from mountain range's base

Step 4: Calculate $\text{area}_{\text{total}}$ and $\text{area}_{\text{intact}}$ within each species' elevational range

Example: $s_i = 10 \times (500 / 100) / 2 = 25$ species for mountain range *i* with a_i = 500 m and b_i = 10 bands

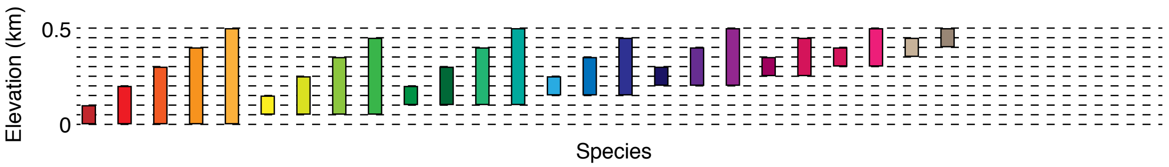

Step 4: Subject species to range shift

Range shift_i (km) = Warming rate_i (˚C) / Lapse rate_i (˚C / km)

Warming $\text{rate}_i = \text{mean}_{\text{Raster}}(\text{mean}_{\text{RasterStack}}(\text{MAT}_{\text{GCM}(1,\ldots,17)} - \text{MAT}_{\text{current}}))_i$,

Lapse rate_i = coefficient of lm(MAT ~ elevation)

MAT = mean annual temperature

Step 5: Calculate $\text{area}_{\text{total}}$ and $\text{area}_{\text{intact}}$ within each species' range following the range shift

Step 6: Calculate $\Delta\text{area}_{\text{total}}$ and $\Delta\text{area}_{\text{intact}}$

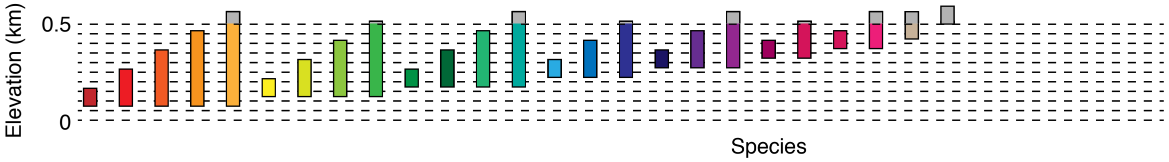

Step 7: Calculate mean and SE $\Delta\text{area}_{\text{total}}$ and $\Delta\text{area}_{\text{intact}}$ across all species per elevational band

Step 8: Calculate proportion of elevational range where $\Delta\text{area}_{\text{intact}} \geq \Delta\text{area}_{\text{total}}$

Supplementary Figure 9. Descriptive and schematic overview of hypothetical species range shift modeling procedure. The example considers a mountain range with a 500 m amplitude extending from sea level to 500 m.

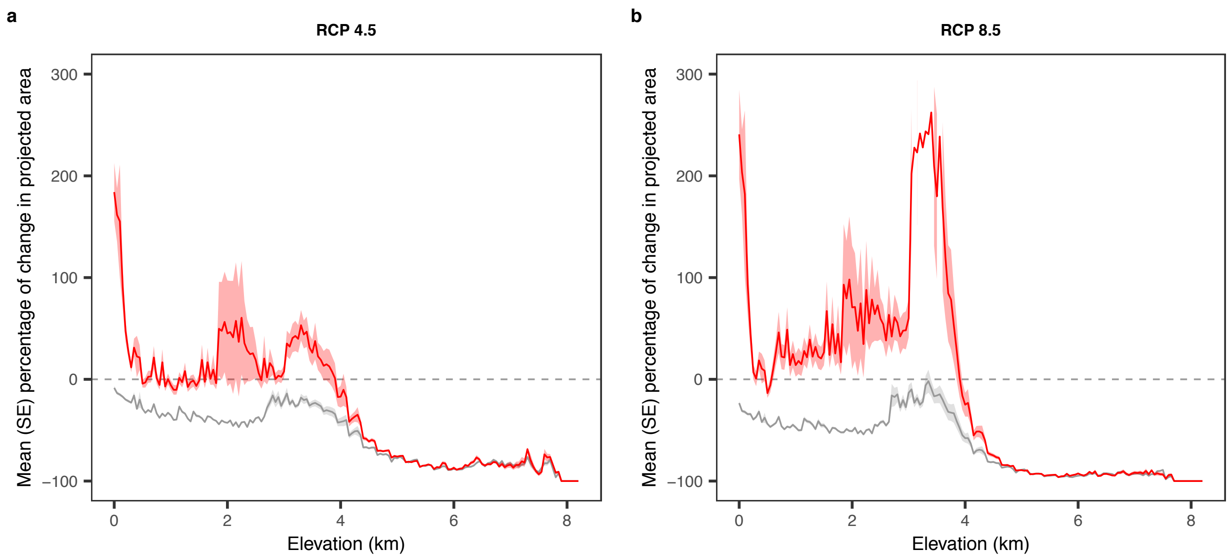

Supplementary Figure 10. Average projected area changes for species undergoing elevational range shifts at the global scale. Mean (lines) and standard error (shaded regions) percentage of change in projected total (grey lines) and intact (red lines; threshold HFI value = 4) land area across all modeled species and all mountain ranges under RCP 4.5 (**a**) and RCP 8.5 (**b**). See text and Methods for details of modeled range shifts.
