# Supplementary material for: Topography and human pressure in mountain ranges alter expected species responses to climate change
Source: Nat Commun. 2020 Apr 24;11:1974. doi: 10.1038/s41467-020-15881-x (PMC7181879; doi:10.1038/s41467-020-15881-x)
Supplement: Supplementary file 3 — Reporting Summary [file 41467_2020_15881_MOESM3_ESM.pdf]

nature research

Corresponding author(s): DBPR
Last updated by author(s): 03/23/2020

Reporting Summary

Nature Research wishes to improve the reproducibility of the work that we publish. This form provides structure for consistency and transparency in reporting. For further information on Nature Research policies, see Authors & Referees and the Editorial Policy Checklist .

Statistics

For all statistical analyses, confirm that the following items are present in the figure legend, table legend, main text, or Methods section.

n/a	Confirmed	
☐	☒	The exact sample size (*n*) for each experimental group/condition, given as a discrete number and unit of measurement
☒	☐	A statement on whether measurements were taken from distinct samples or whether the same sample was measured repeatedly
☐	☒	The statistical test(s) used AND whether they are one- or two-sided<br>*Only common tests should be described solely by name; describe more complex techniques in the Methods section.*
☒	☐	A description of all covariates tested
☐	☒	A description of any assumptions or corrections, such as tests of normality and adjustment for multiple comparisons
☐	☒	A full description of the statistical parameters including central tendency (e.g. means) or other basic estimates (e.g. regression coefficient) AND variation (e.g. standard deviation) or associated estimates of uncertainty (e.g. confidence intervals)
☐	☒	For null hypothesis testing, the test statistic (e.g. *F*, *t*, *r*) with confidence intervals, effect sizes, degrees of freedom and *P* value noted<br>*Give P values as exact values whenever suitable.*
☒	☐	For Bayesian analysis, information on the choice of priors and Markov chain Monte Carlo settings
☒	☐	For hierarchical and complex designs, identification of the appropriate level for tests and full reporting of outcomes
☒	☐	Estimates of effect sizes (e.g. Cohen's *d*, Pearson's *r*), indicating how they were calculated

Our web collection on statistics for biologists contains articles on many of the points above.

Software and code

Policy information about availability of computer code

Data collection	Mountain range data: GMBA version 1.1. Elevation data: SRTM version 3. Human footprint: Venter et al. (2016), 2009 layer. WorldClim current and future mean annual temperature data (versions 1.4 and 2.0 used).
Data analysis	Custom code written in R version 3.6.1. Sample code and data were uploaded with the separate code checklist.

For manuscripts utilizing custom algorithms or software that are central to the research but not yet described in published literature, software must be made available to editors/reviewers. We strongly encourage code deposition in a community repository (e.g. GitHub). See the Nature Research guidelines for submitting code & software for further information.

Data

Policy information about availability of data

All manuscripts must include a data availability statement. This statement should provide the following information, where applicable:

- Accession codes, unique identifiers, or web links for publicly available datasets
- A list of figures that have associated raw data
- A description of any restrictions on data availability

GMBA, SRTM elevation, WorldClim, and human footprint data are freely available. Links to datasets are included in the Methods and References sections in the paper.

Field-specific reporting

Please select the one below that is the best fit for your research. If you are not sure, read the appropriate sections before making your selection.

Ecological, evolutionary & environmental sciences study design

All studies must disclose on these points even when the disclosure is negative.

Study description	This study analyzes patterns of topography in mountain ranges while accounting for human pressure. The study then simulates elevational range shifts for a suite of hypothetical species that vary by elevational range size and shows how the area of occupancy changes, with and without accounting for human pressure
Research sample	The study analyzes all major mountain ranges on earth (1,010) using an exhaustive dataset provided by the Global Mountain Biodiversity Assessment.
Sampling strategy	Complete analysis of all mountain ranges
Data collection	Data is previously published and freely available as noted above.
Timing and spatial scale	Spatial scale is global. Human pressure reflects conditions circa 2009.
Data exclusions	No data were excluded.
Reproducibility	Computer code will be made available.
Randomization	N/A
Blinding	N/A

Did the study involve field work? ☐ Yes ☒ No

Reporting for specific materials, systems and methods

We require information from authors about some types of materials, experimental systems and methods used in many studies. Here, indicate whether each material, system or method listed is relevant to your study. If you are not sure if a list item applies to your research, read the appropriate section before selecting a response.

Materials & experimental systems

n/a	Involved in the study
☒	☐ Antibodies
☒	☐ Eukaryotic cell lines
☒	☐ Palaeontology
☒	☐ Animals and other organisms
☒	☐ Human research participants
☒	☐ Clinical data

Methods

n/a	Involved in the study
☒	☐ ChIP-seq
☒	☐ Flow cytometry
☒	☐ MRI-based neuroimaging
