# Supplementary material for: Topography and human pressure in mountain ranges alter expected species responses to climate change
Source: Nat Commun. 2020 Apr 24;11:1974. doi: 10.1038/s41467-020-15881-x (PMC7181879; doi:10.1038/s41467-020-15881-x)
Supplement: Supplementary file 4 — Description of Additional Supplementary Files [file 41467_2020_15881_MOESM4_ESM.pdf]

Description of Additional Supplementary Files

File Name: Supplementary Data 1
Description: Plots of mean and standard error percentage of changes in area across all modeled species in two cases, one where species are allowed to occupy all land area (blue lines $\Delta Area_{total}$), and one where species only occupy intact land area (red lines $\Delta Area_{intact}$), for all 1,010 mountain ranges (names and unique ID numbers are given above each range).

File Name: Supplementary Data 2
Description: Density plots of elevation and temperature values extracted from an SRTM digital elevation model and current mean annual temperature from WorldClim v2.0 for all 1,010 mountain ranges (names and unique ID numbers are given above each range; warmer tones represent higher data densities). Red lines in each plot are linear fits to the data; the slope of this line is the mountain-range specific lapse rate (see Supplementary Figure 5a).

File Name: Supplementary Software 1
Description: R scripts for performing the mountain classification analysis and modeling elevational range shifts for an example mountain range are available as Supplementary Software.
