# Supplementary figures and images for: Topography and human pressure in mountain ranges alter expected species responses to climate change
Source: Nat Commun. 2020 Apr 24;11:1974. doi: 10.1038/s41467-020-15881-x (PMC7181879; doi:10.1038/s41467-020-15881-x)

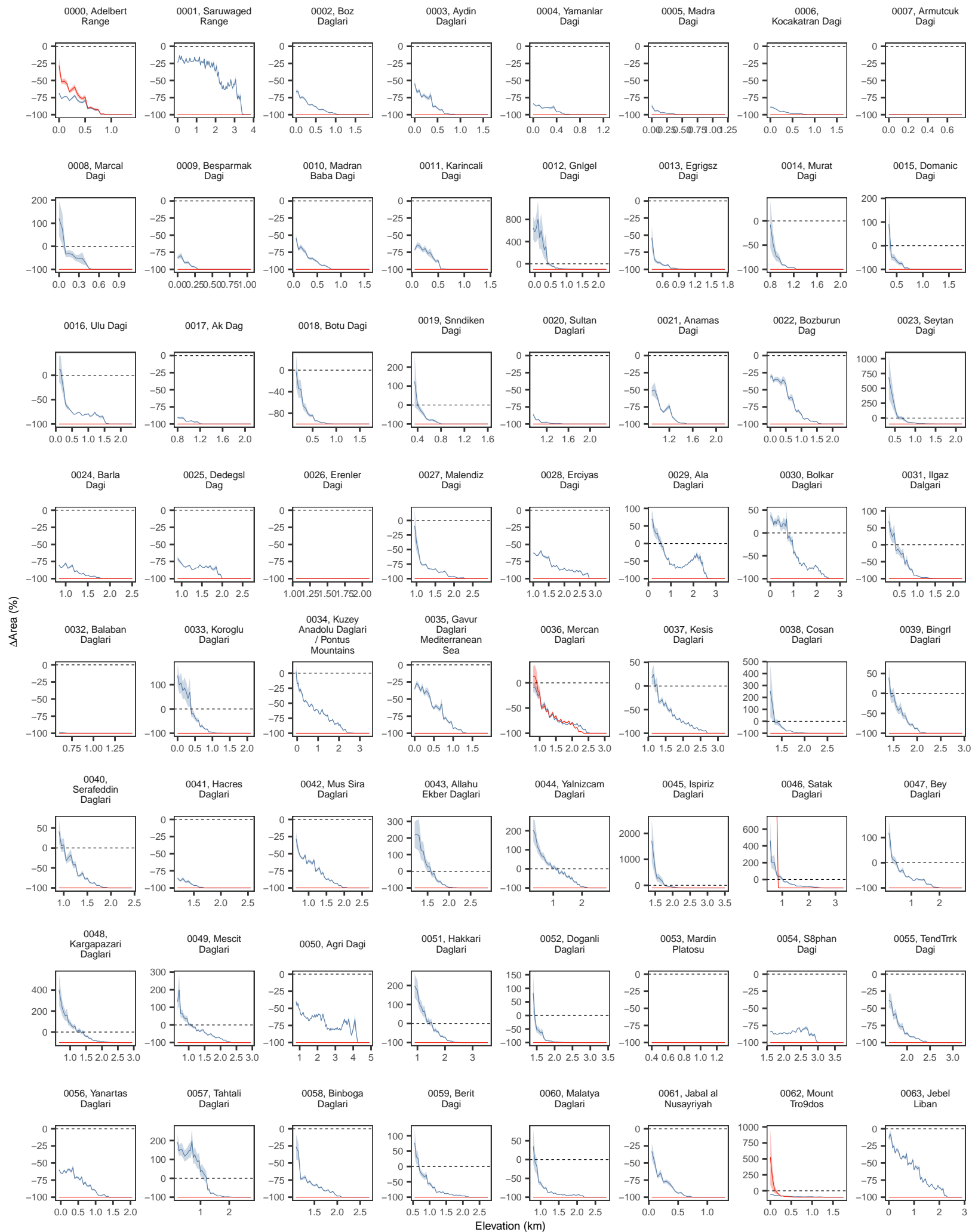

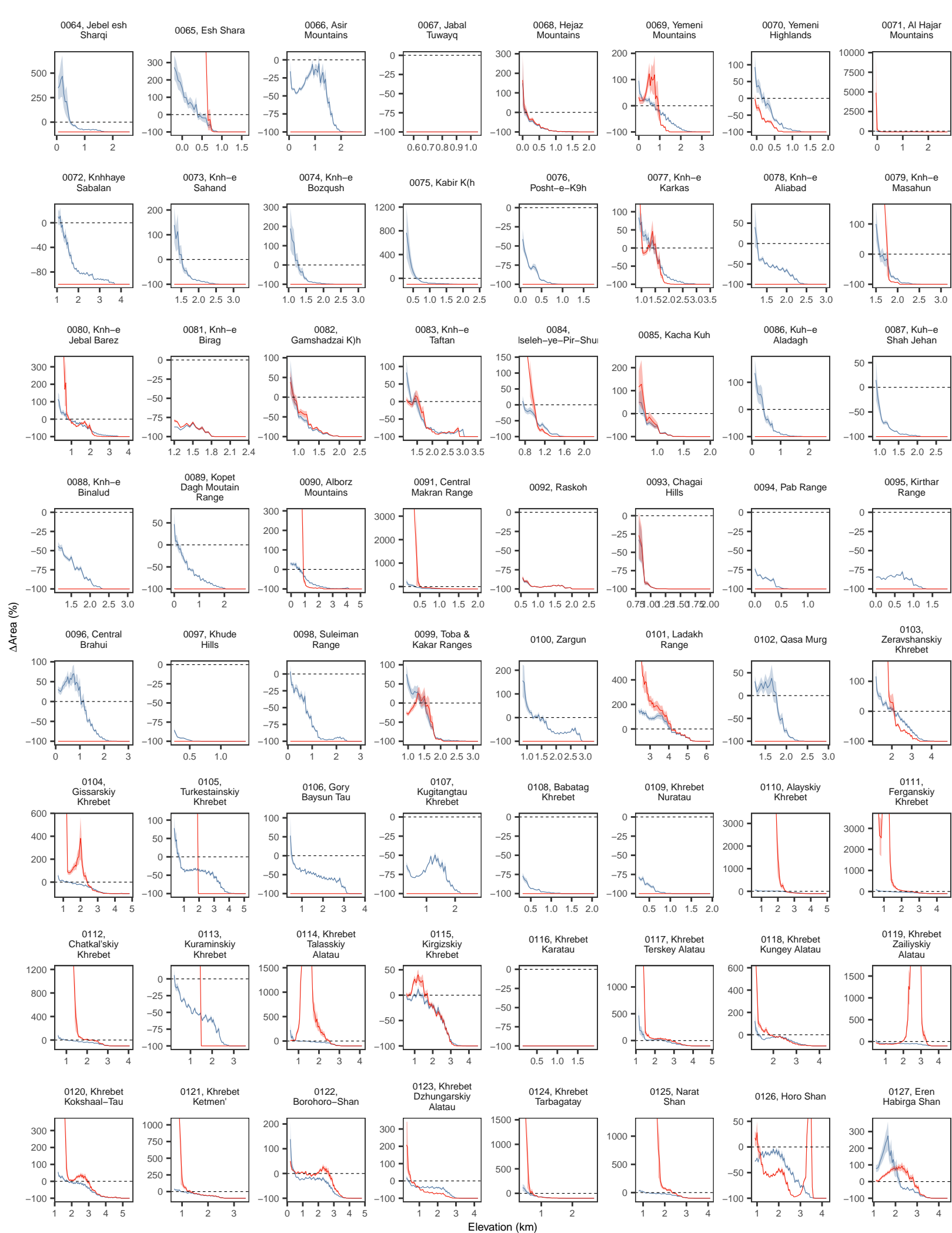

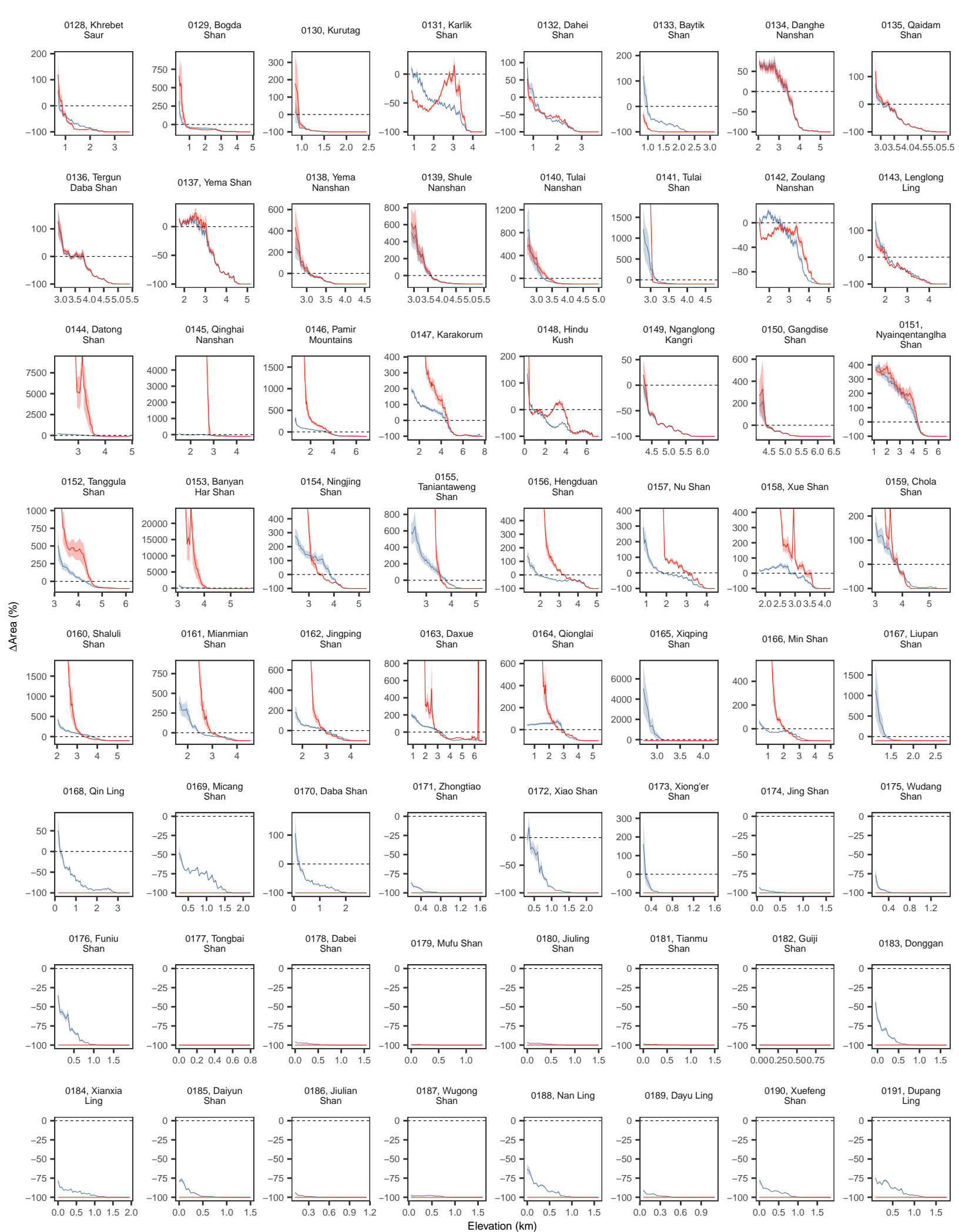

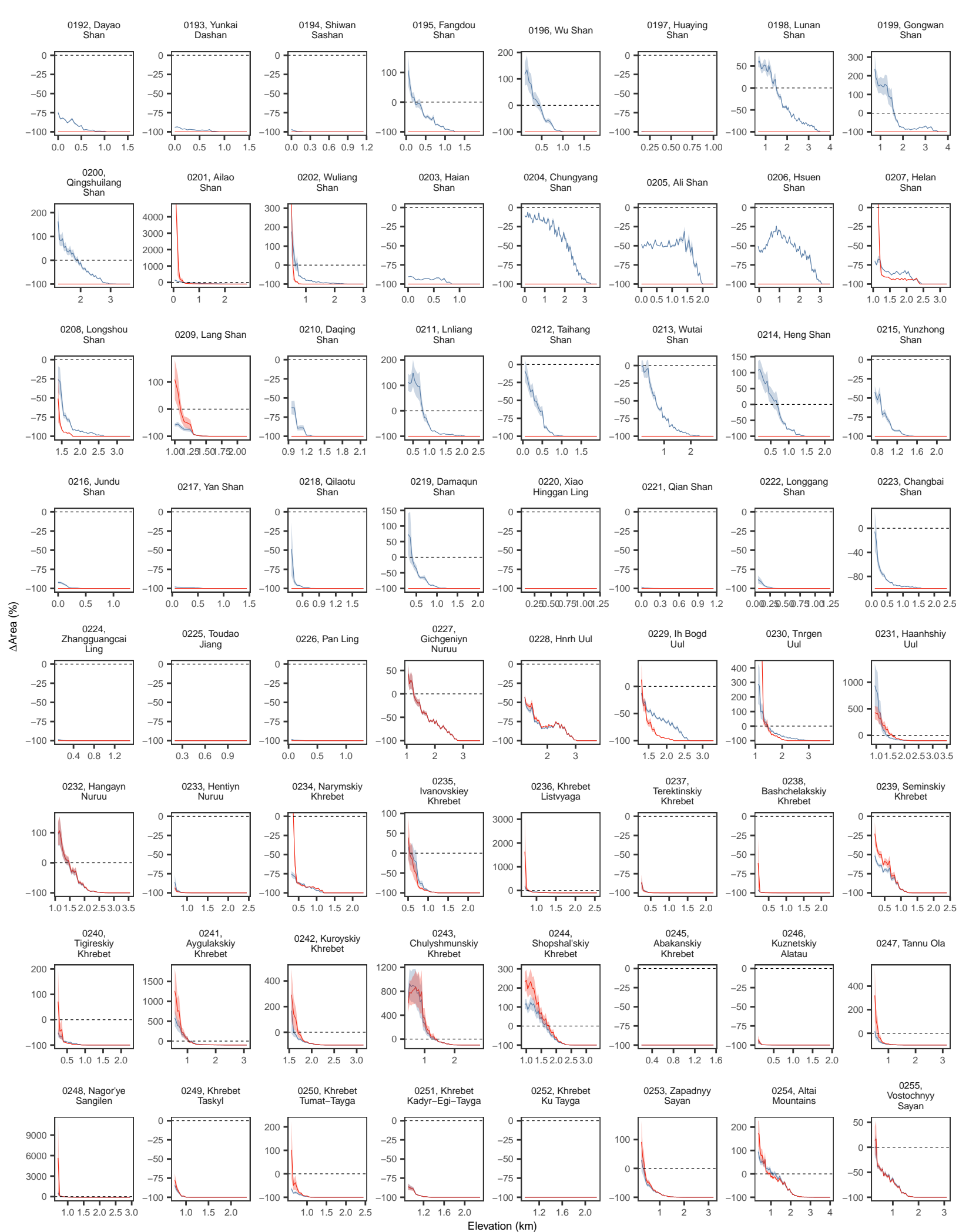

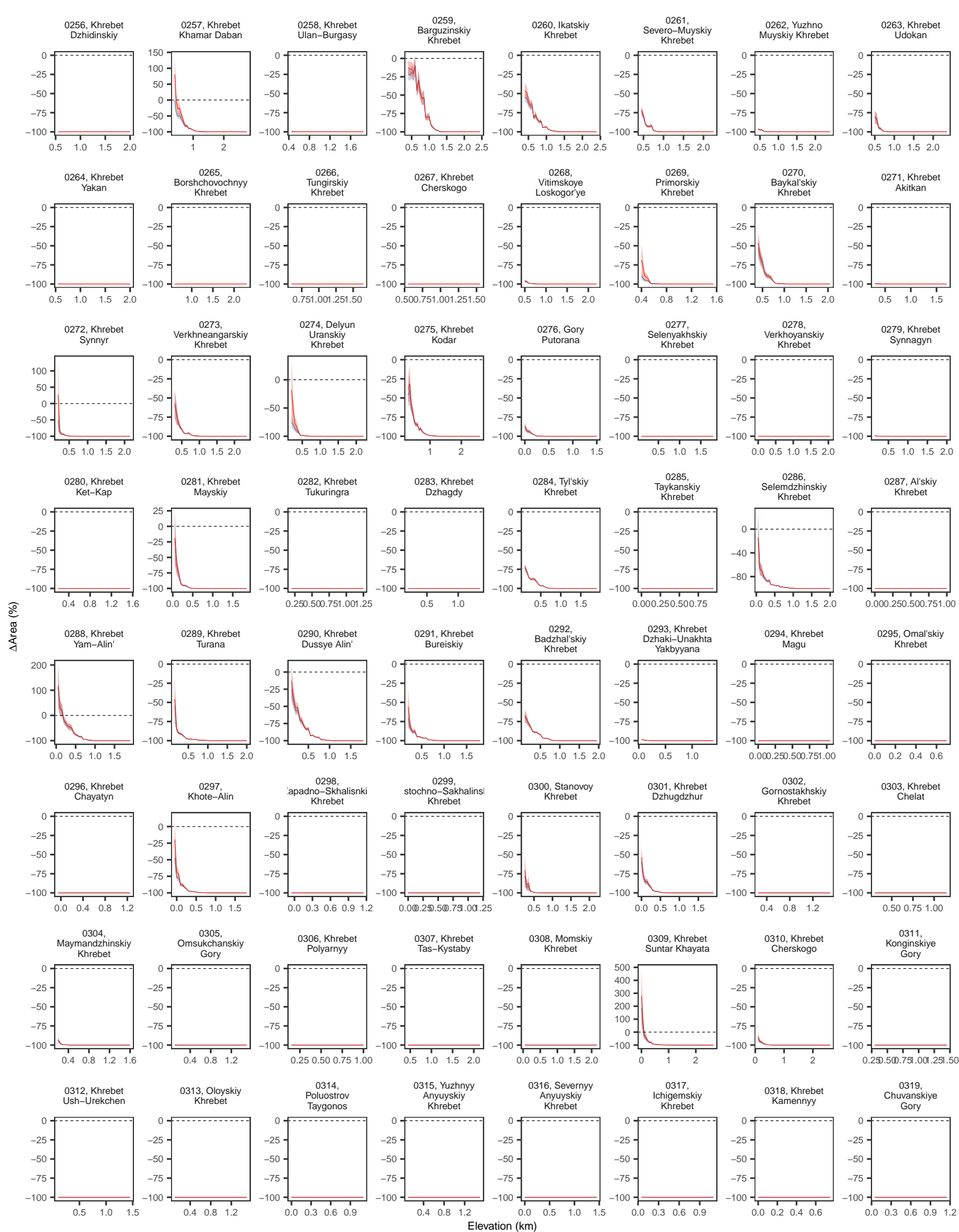

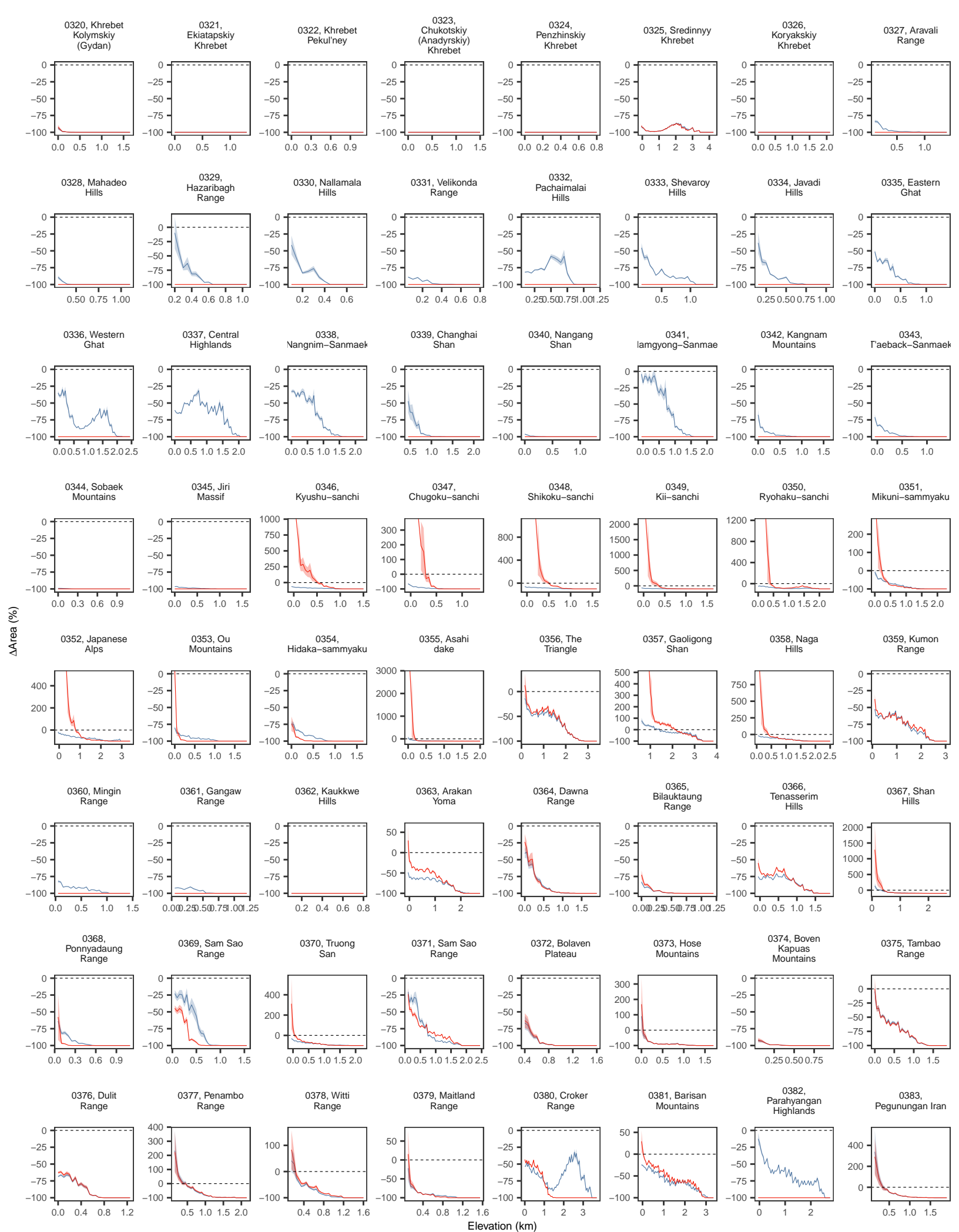

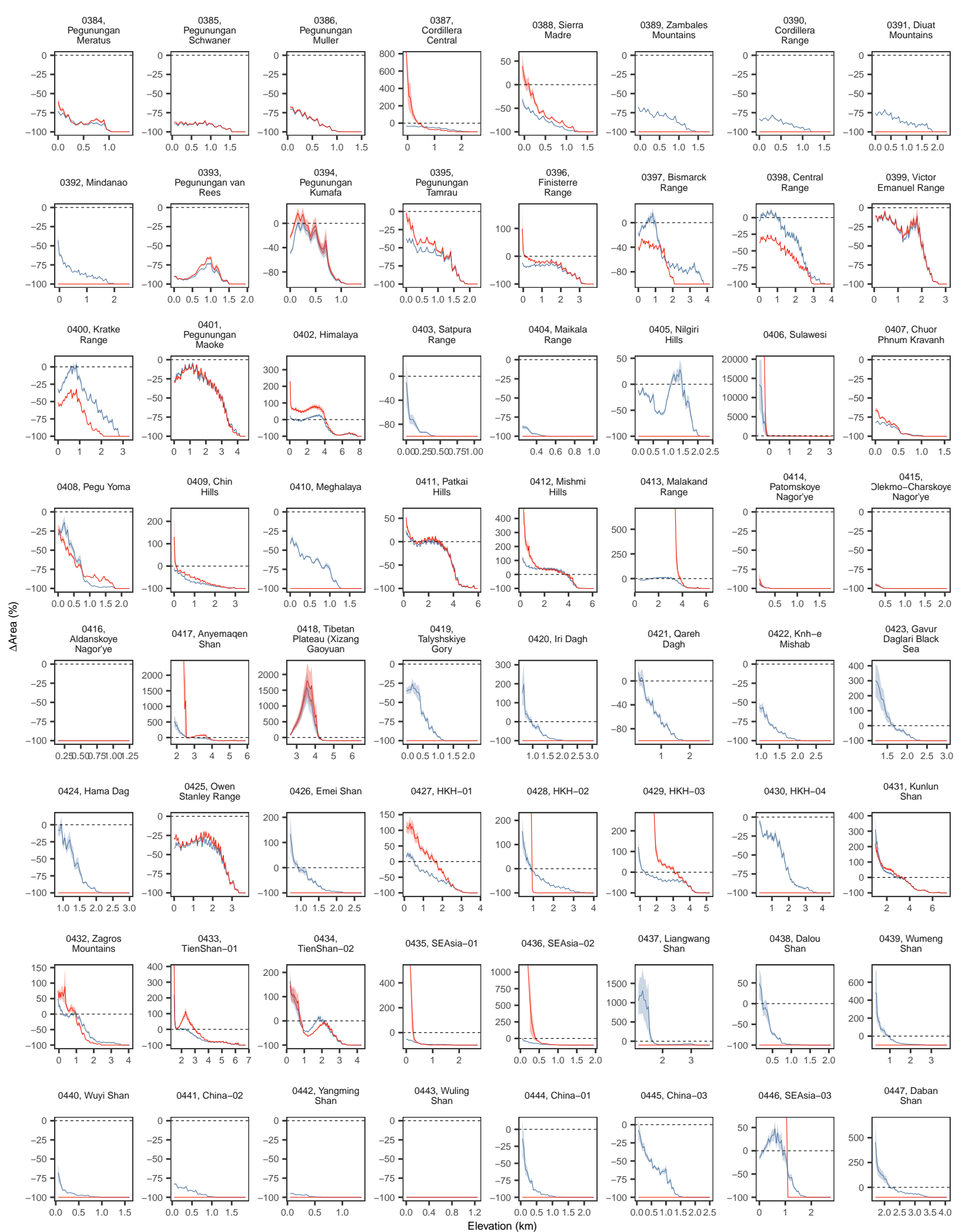

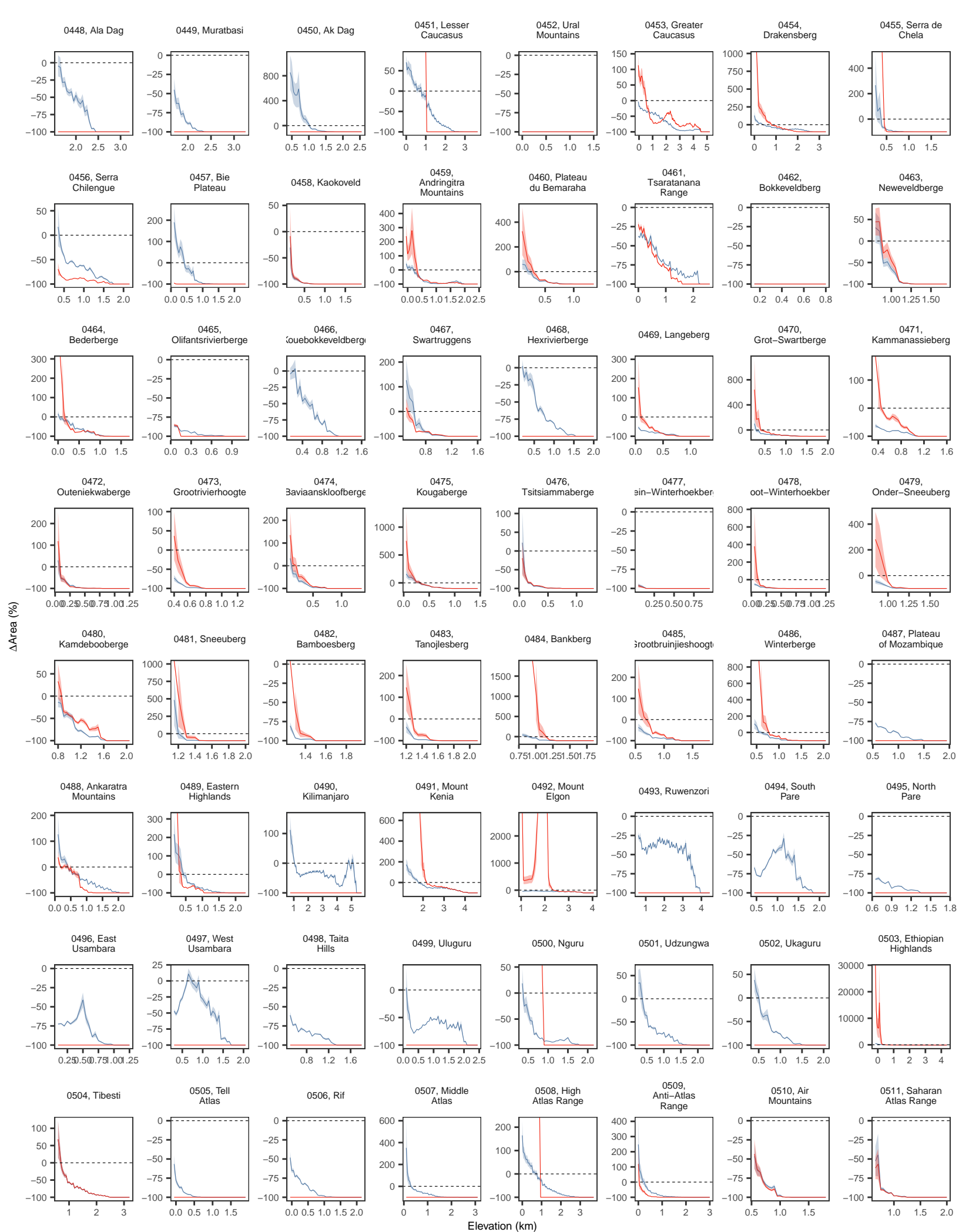

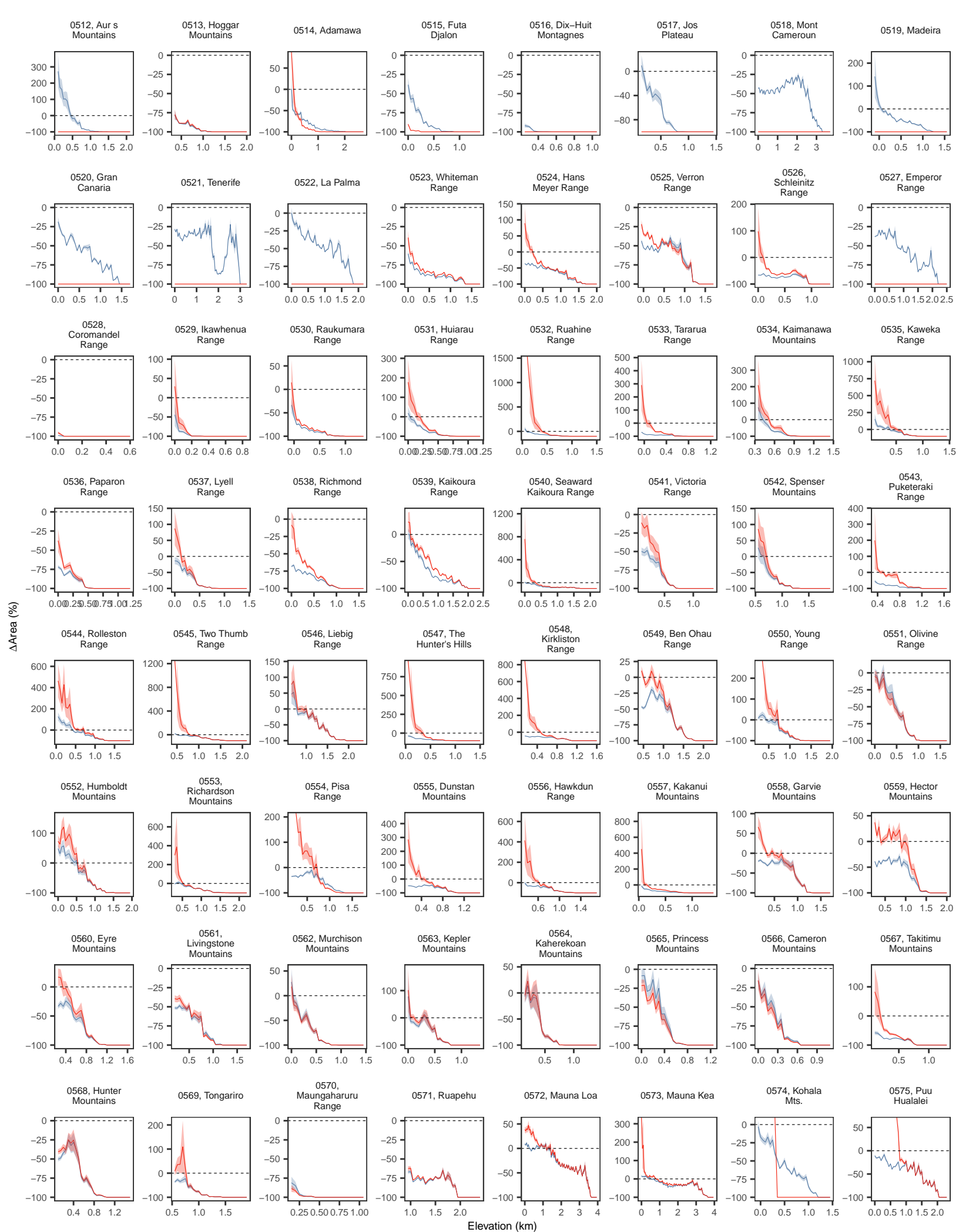

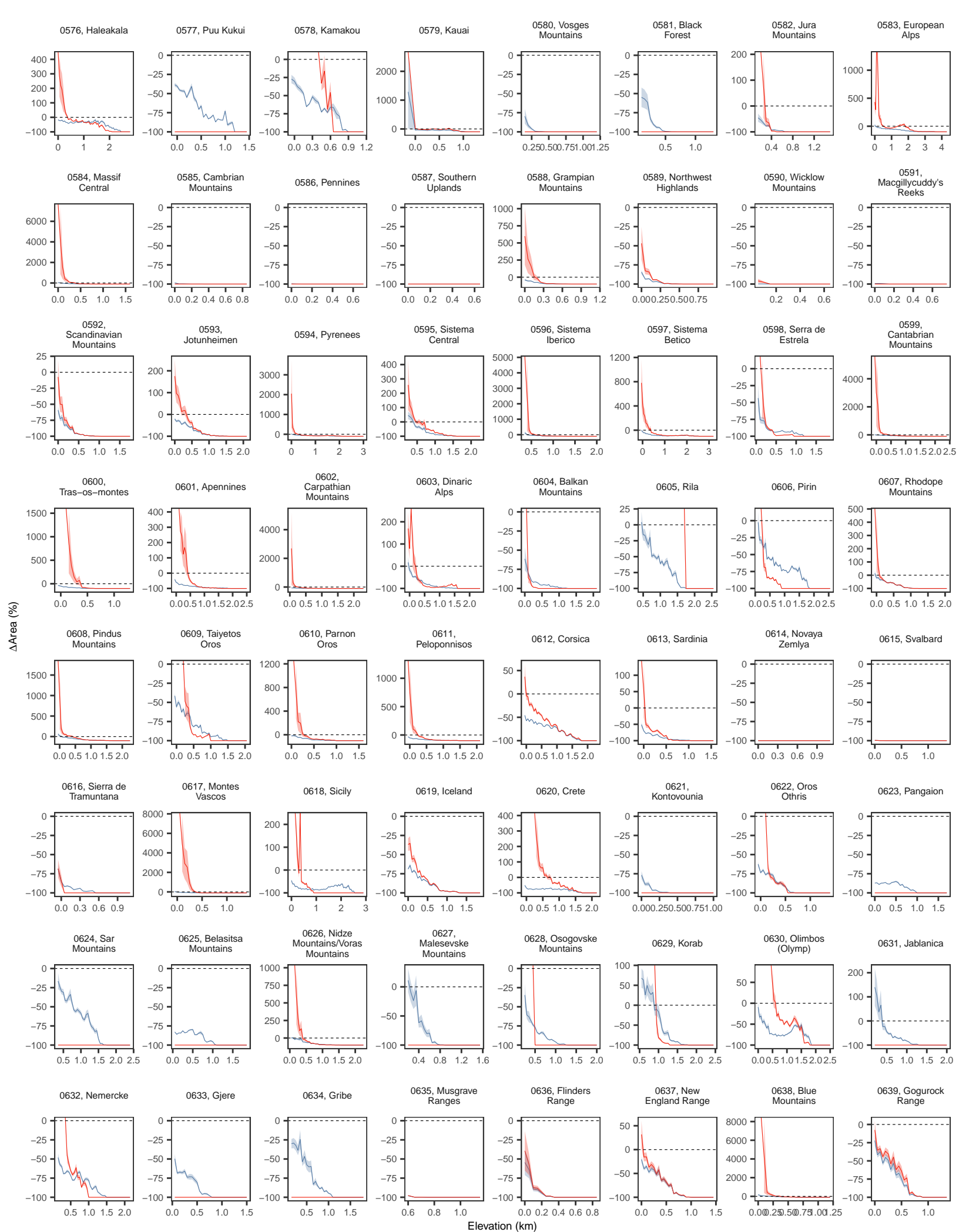

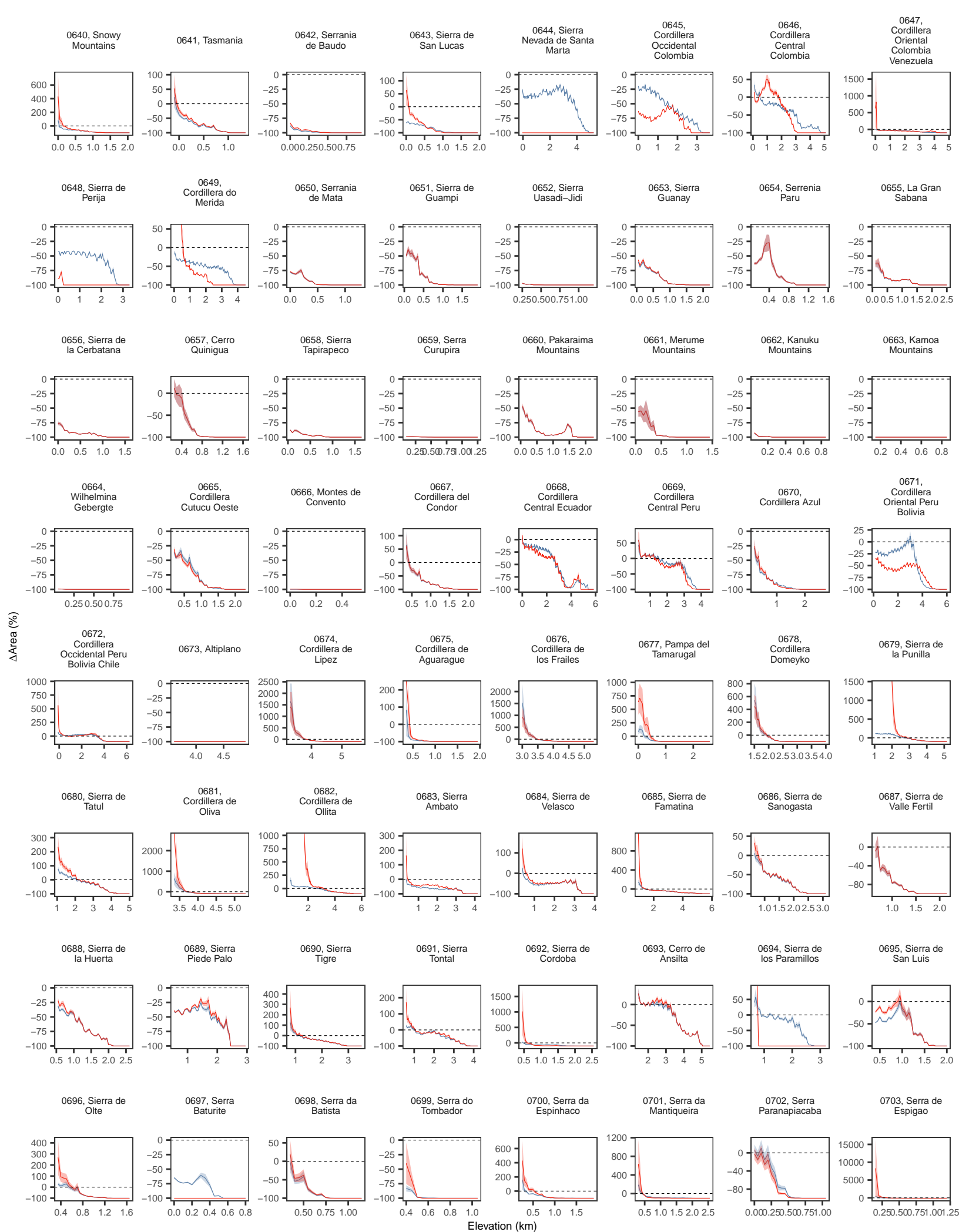

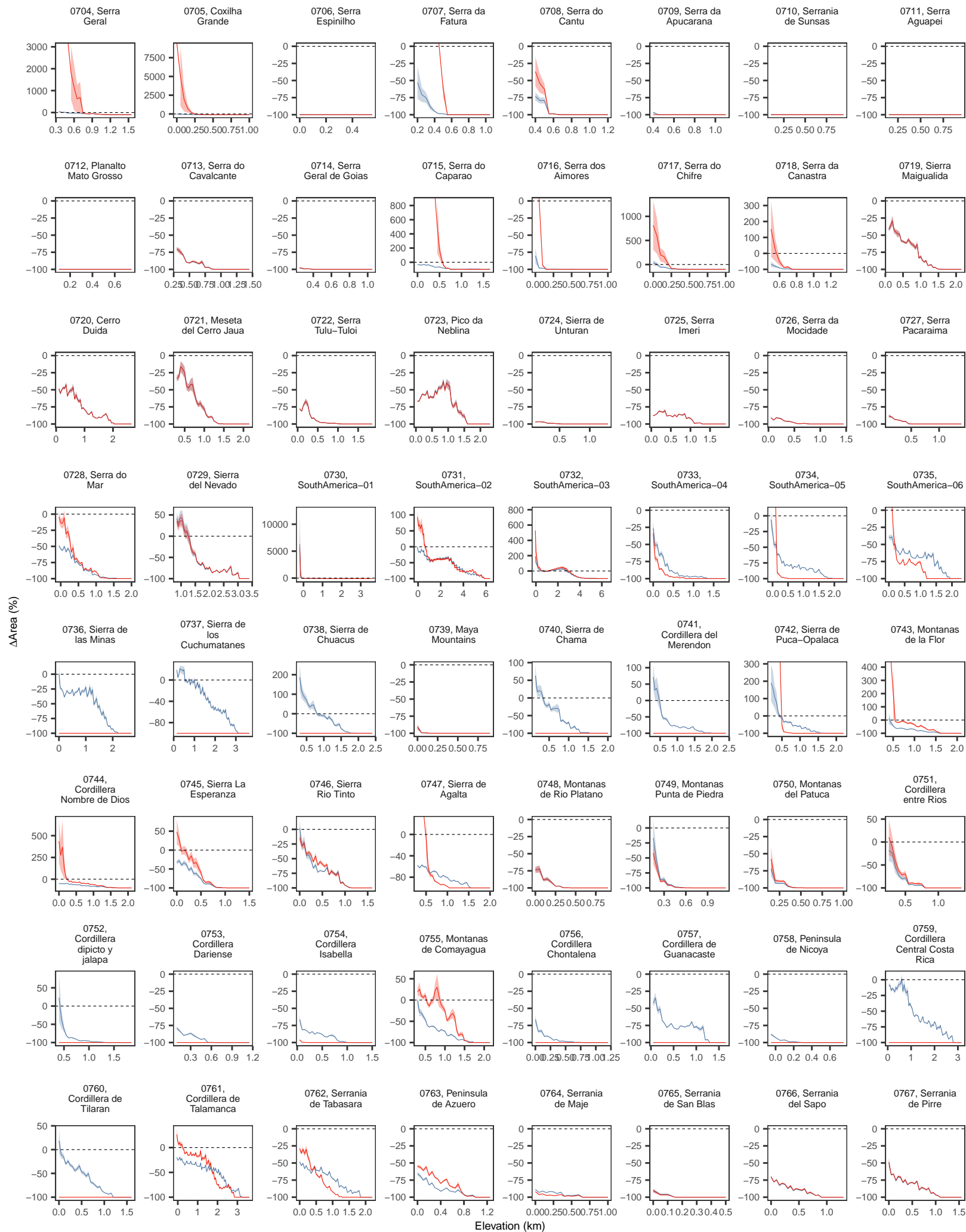

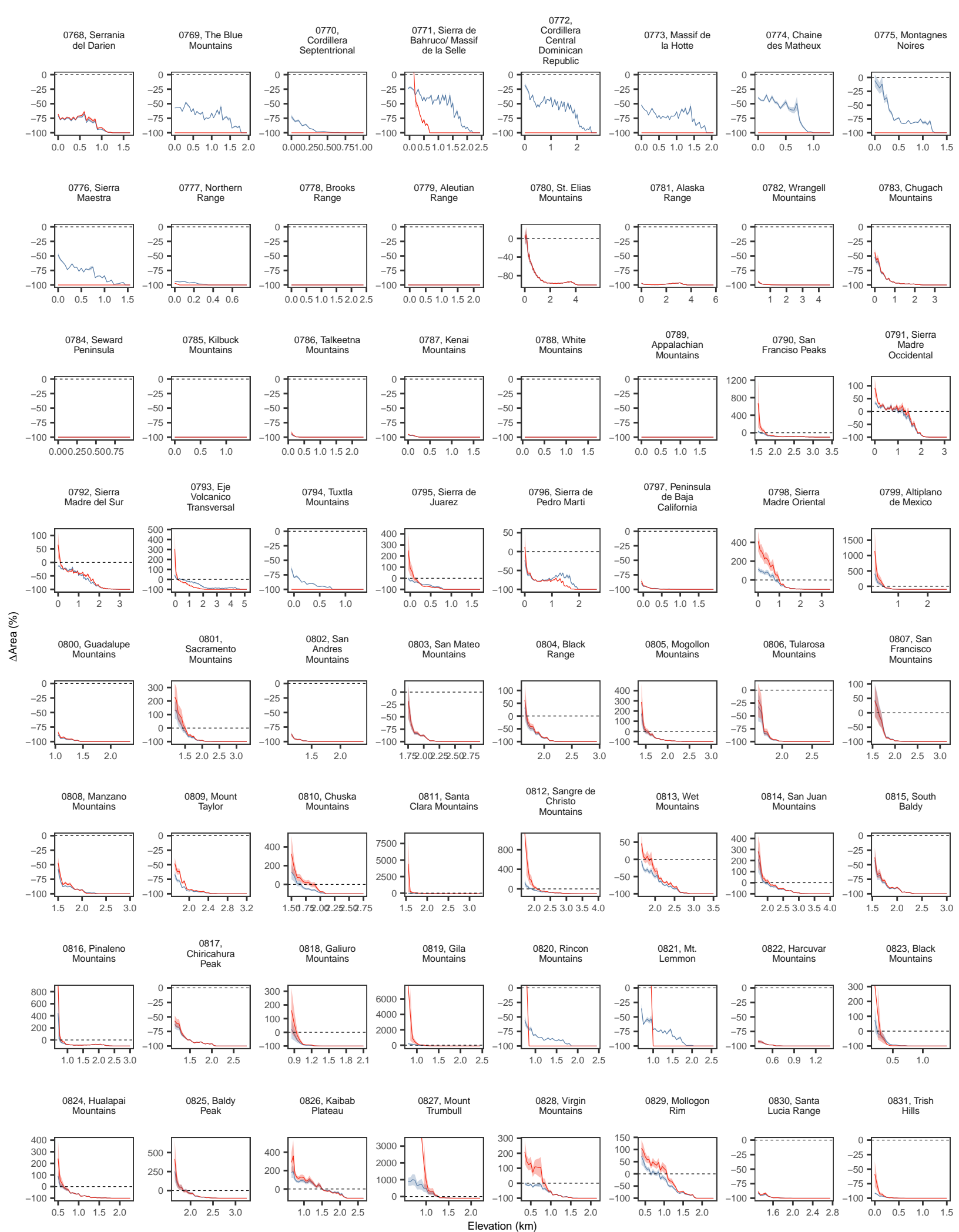

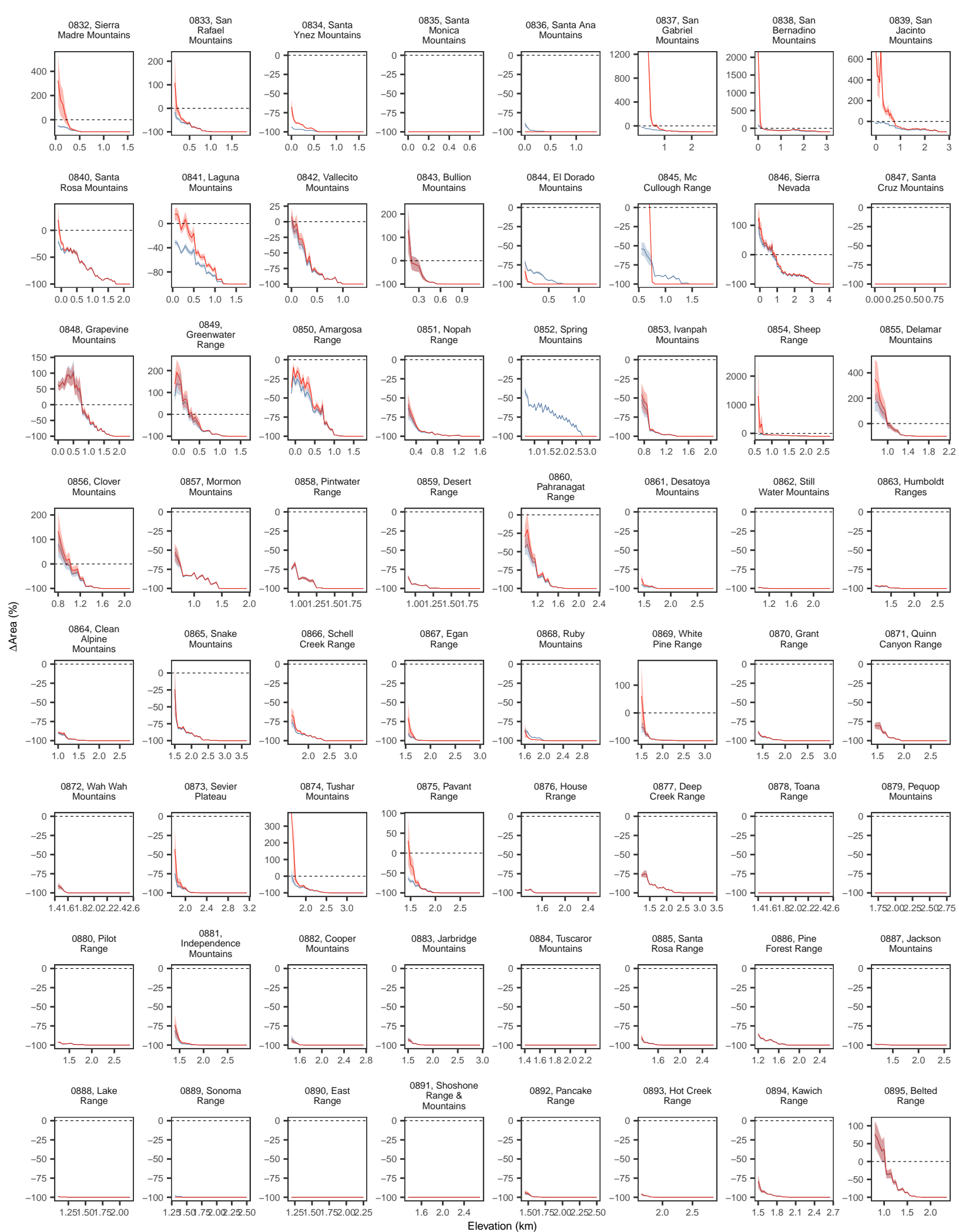

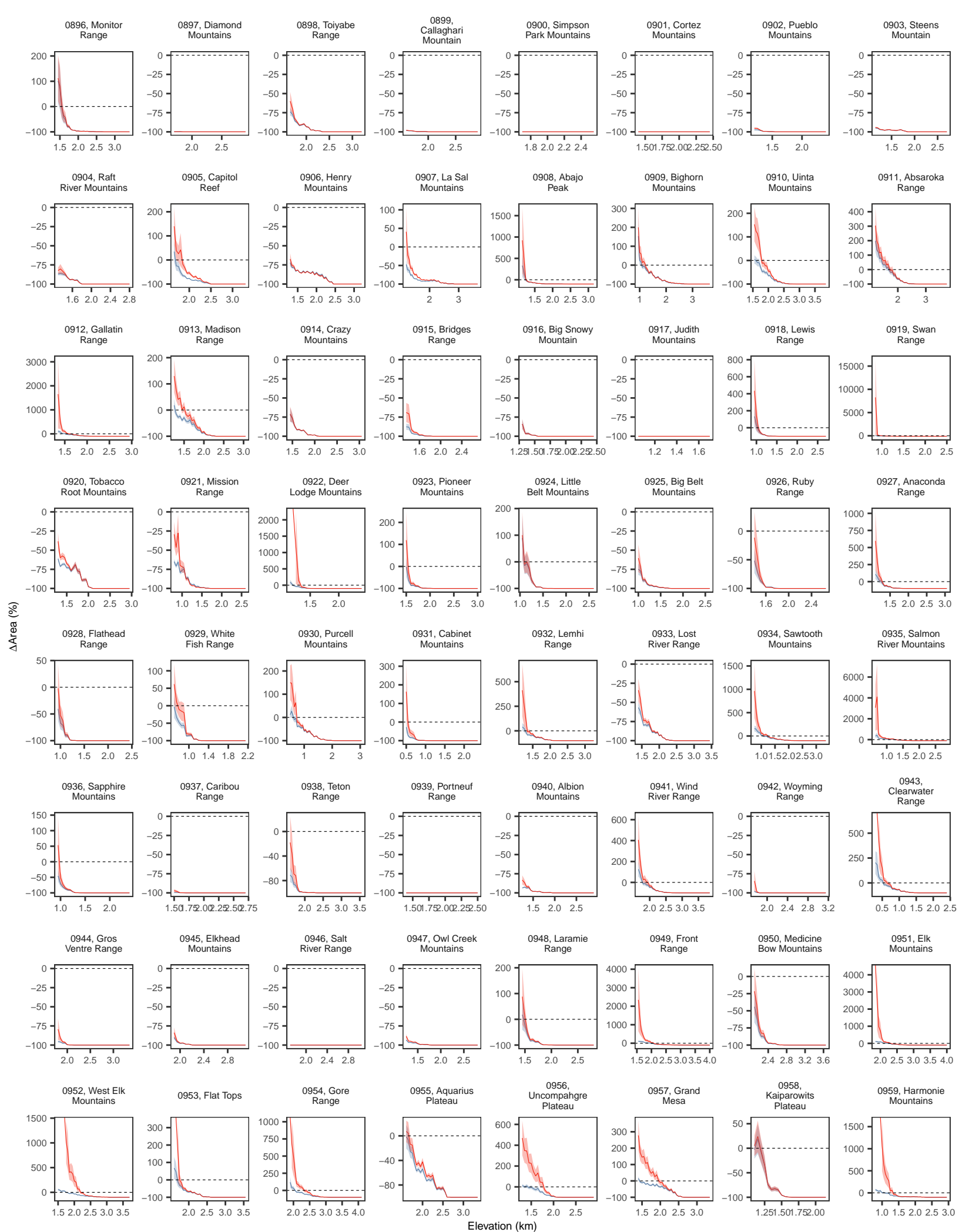

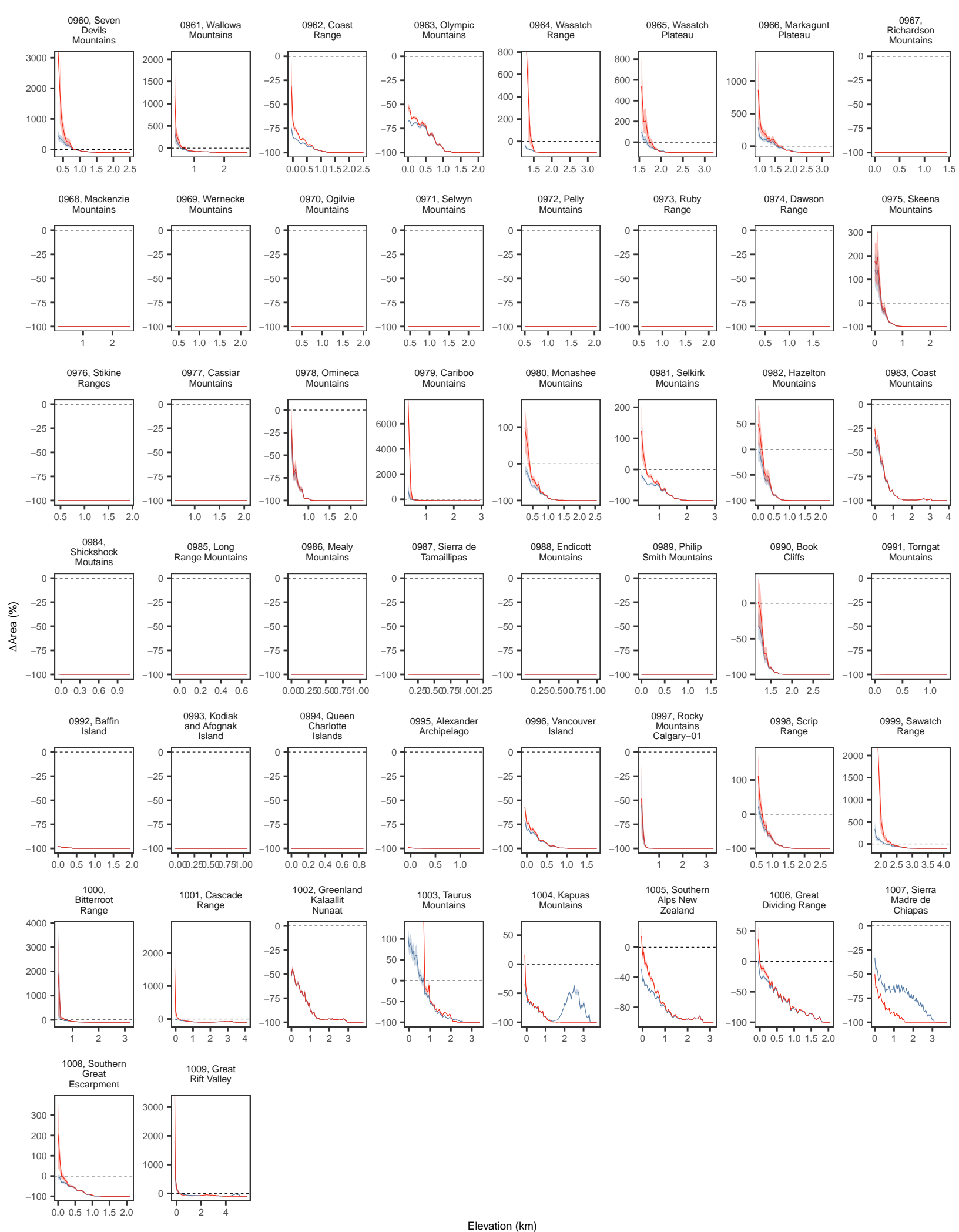

Supplement: Supplementary file 5 — Supplementary Data 1 [file 41467_2020_15881_MOESM5_ESM.pdf]

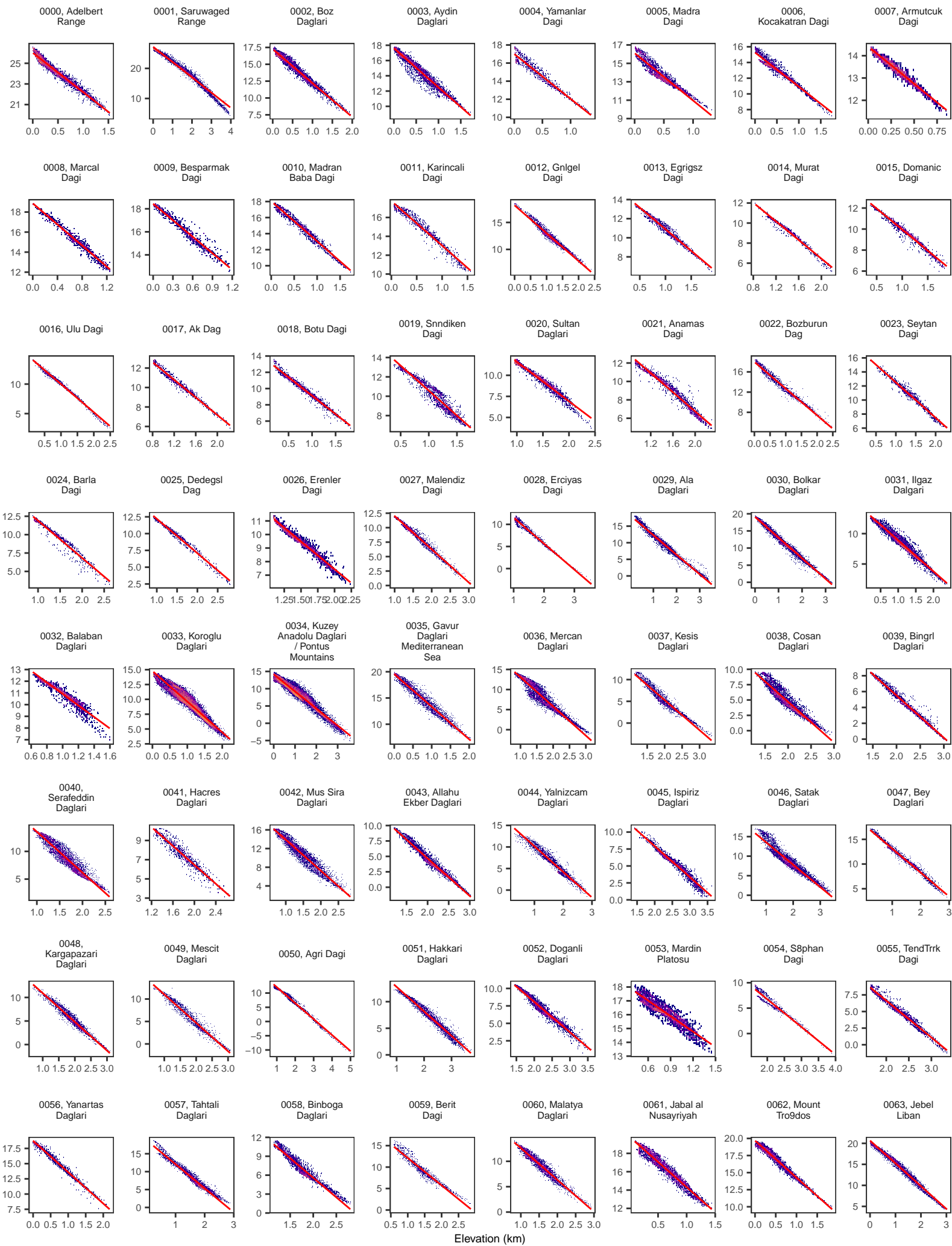

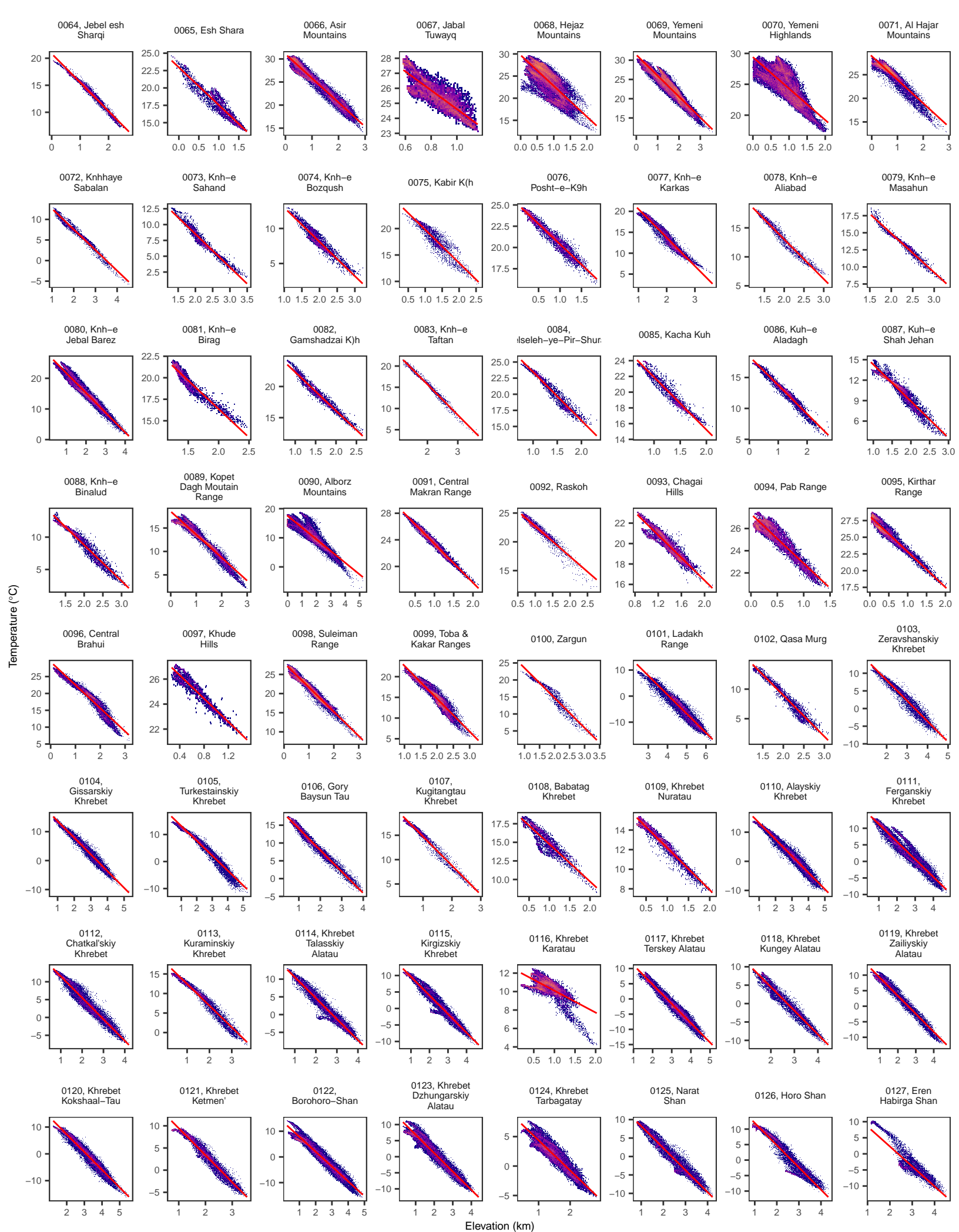

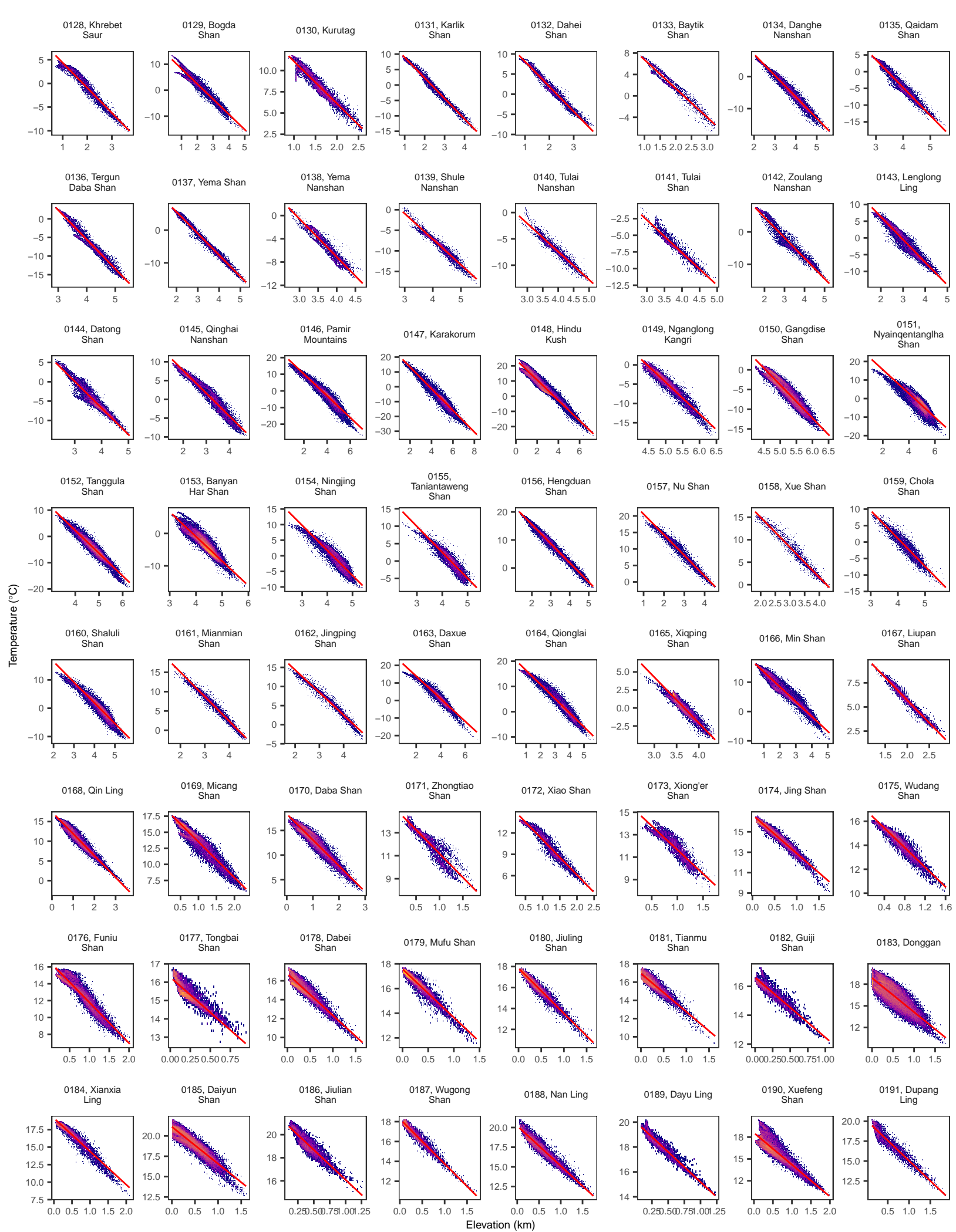

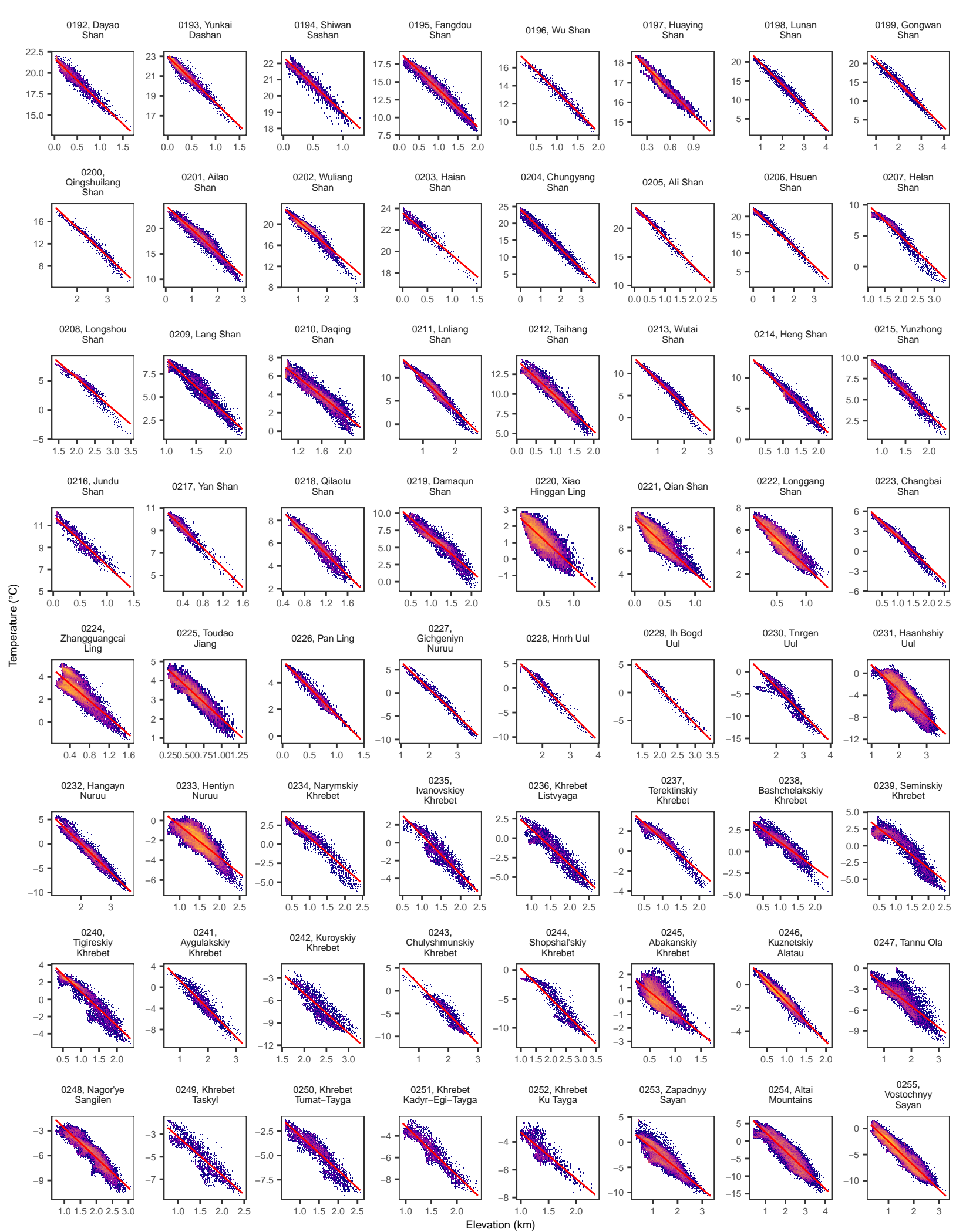

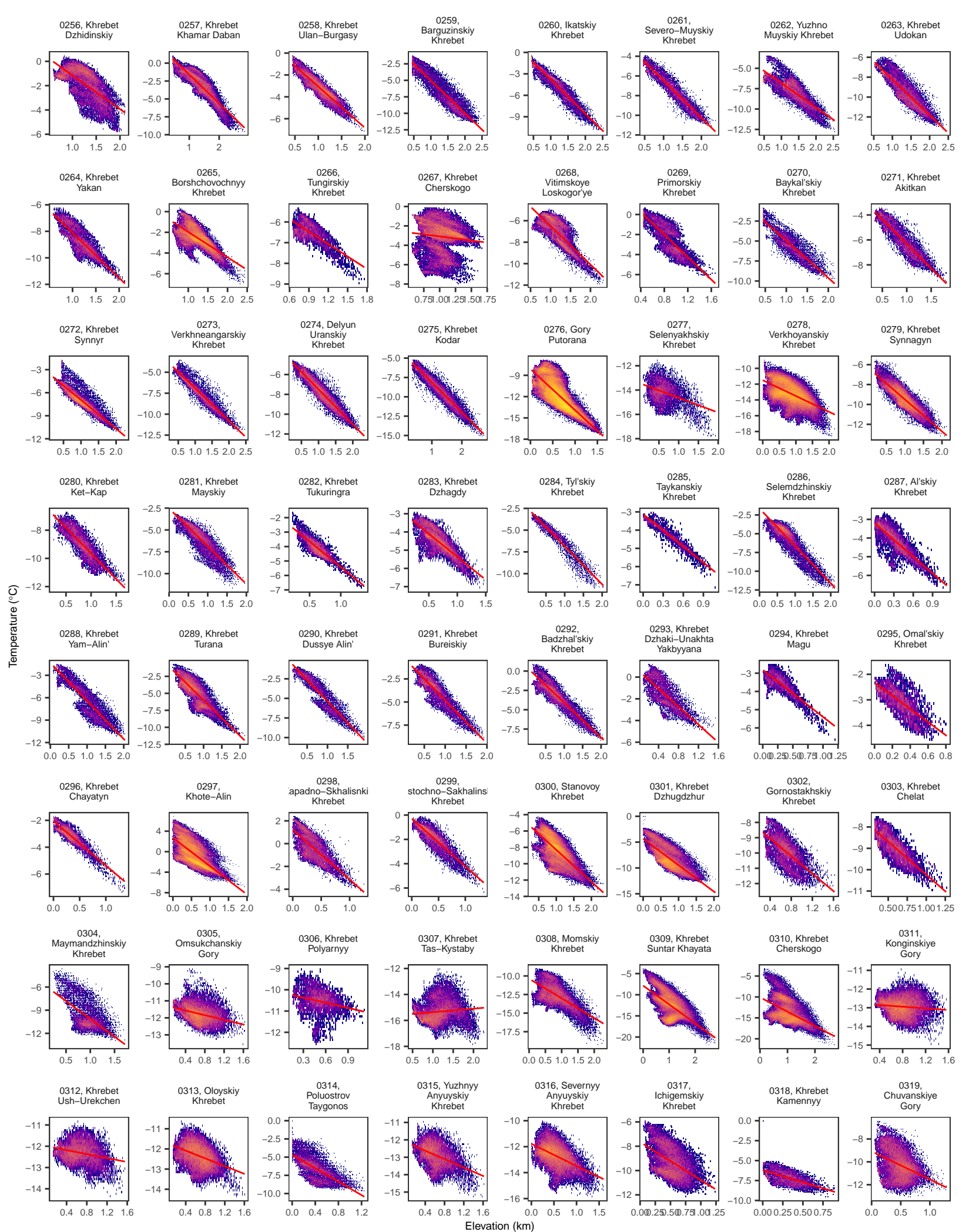

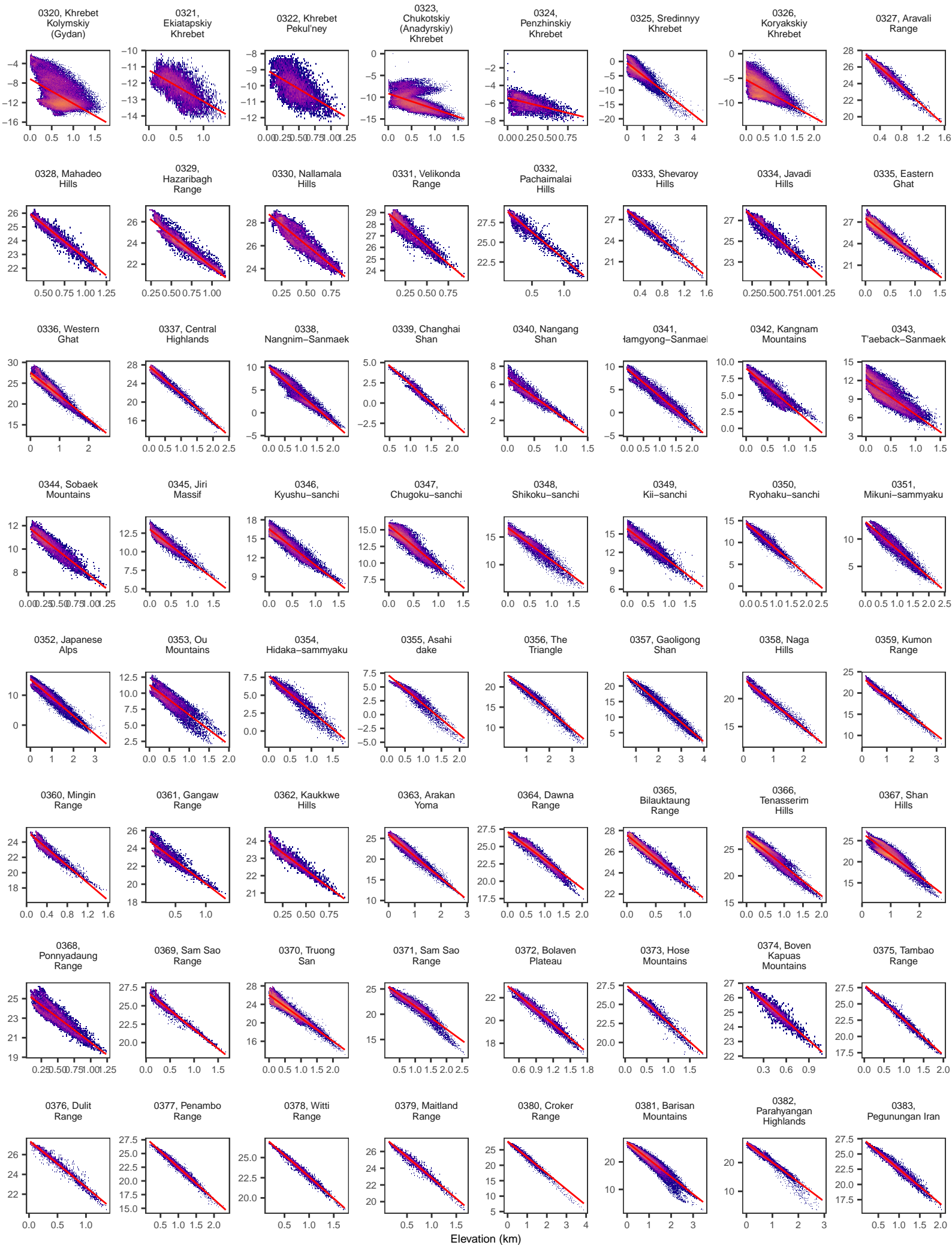

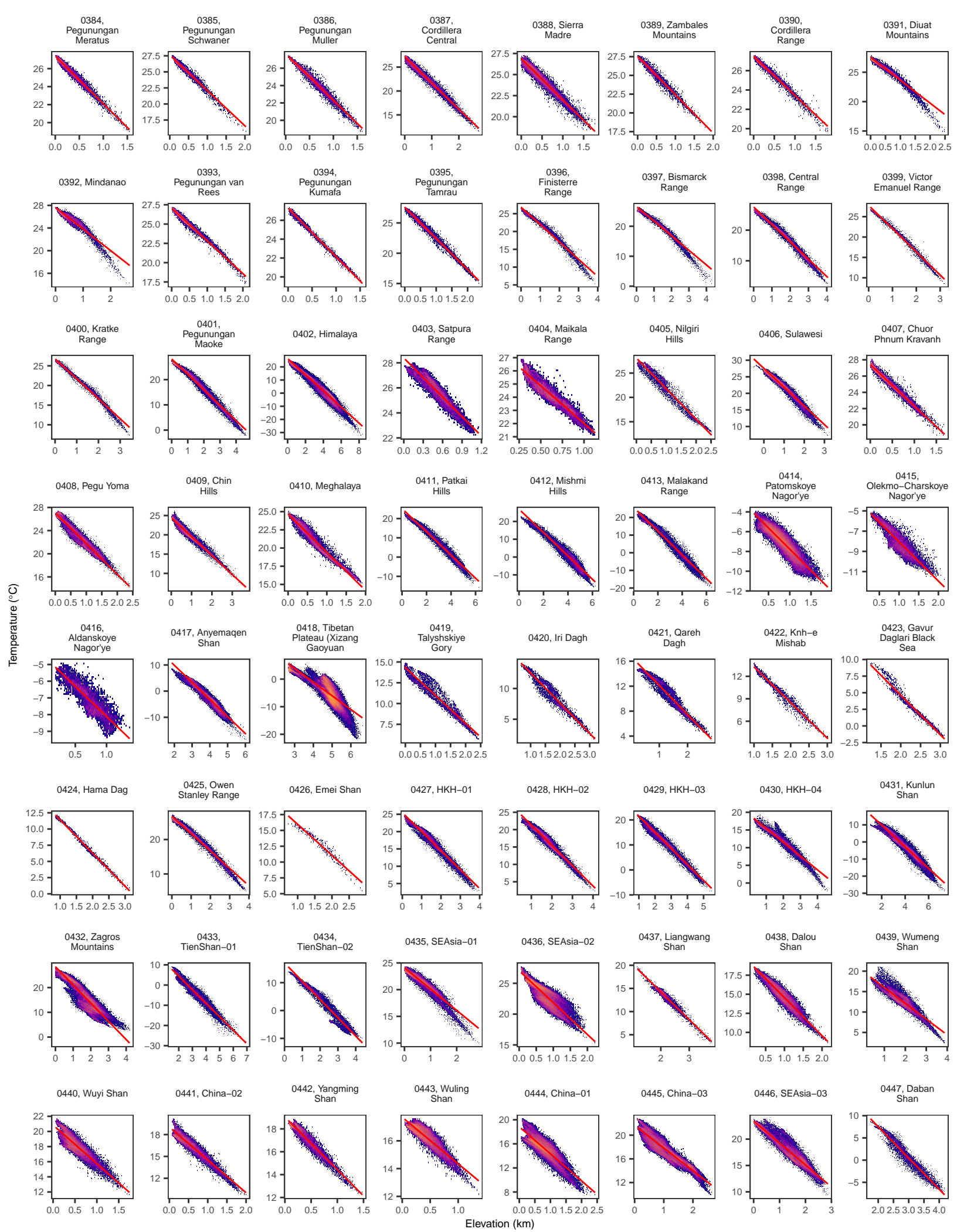

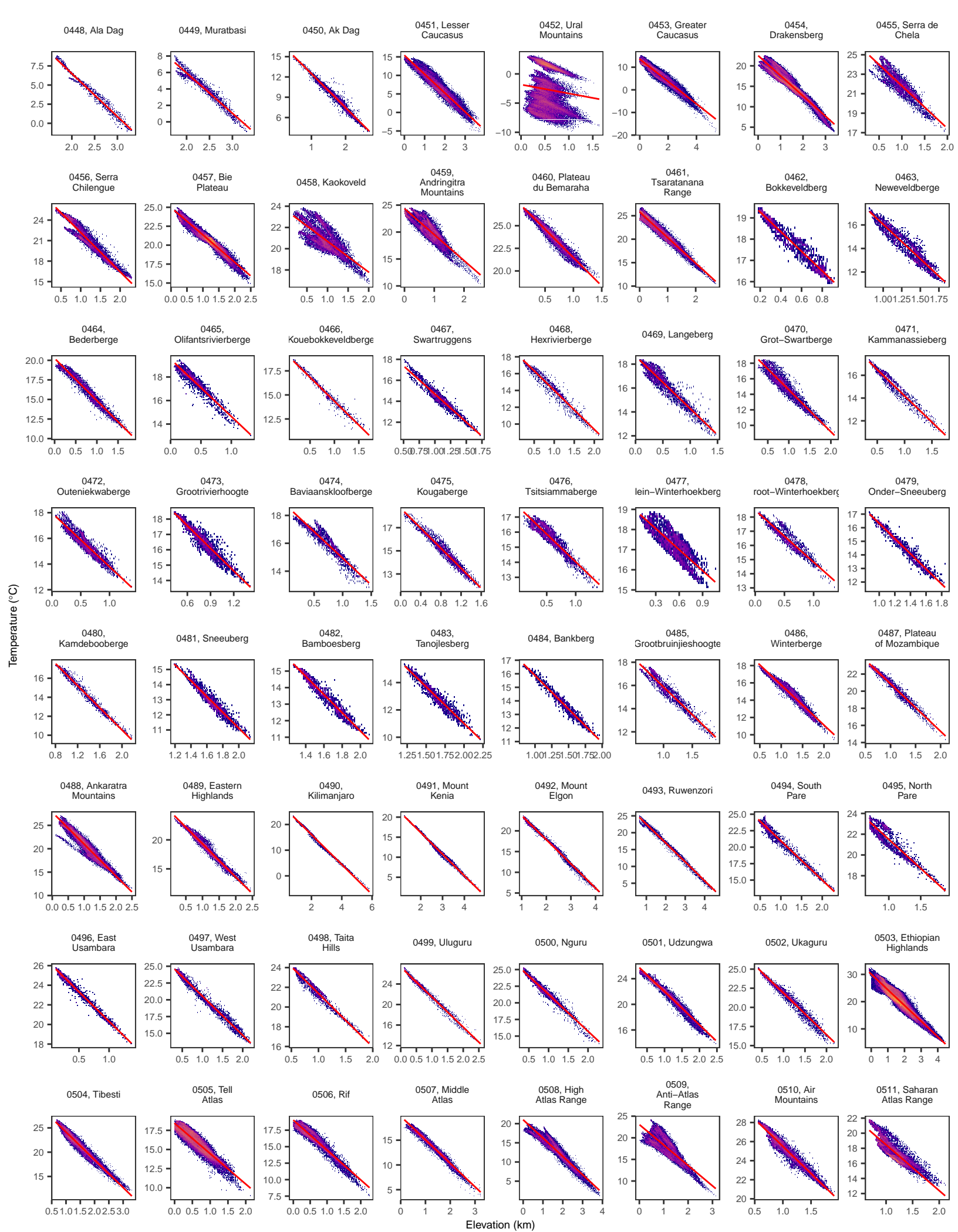

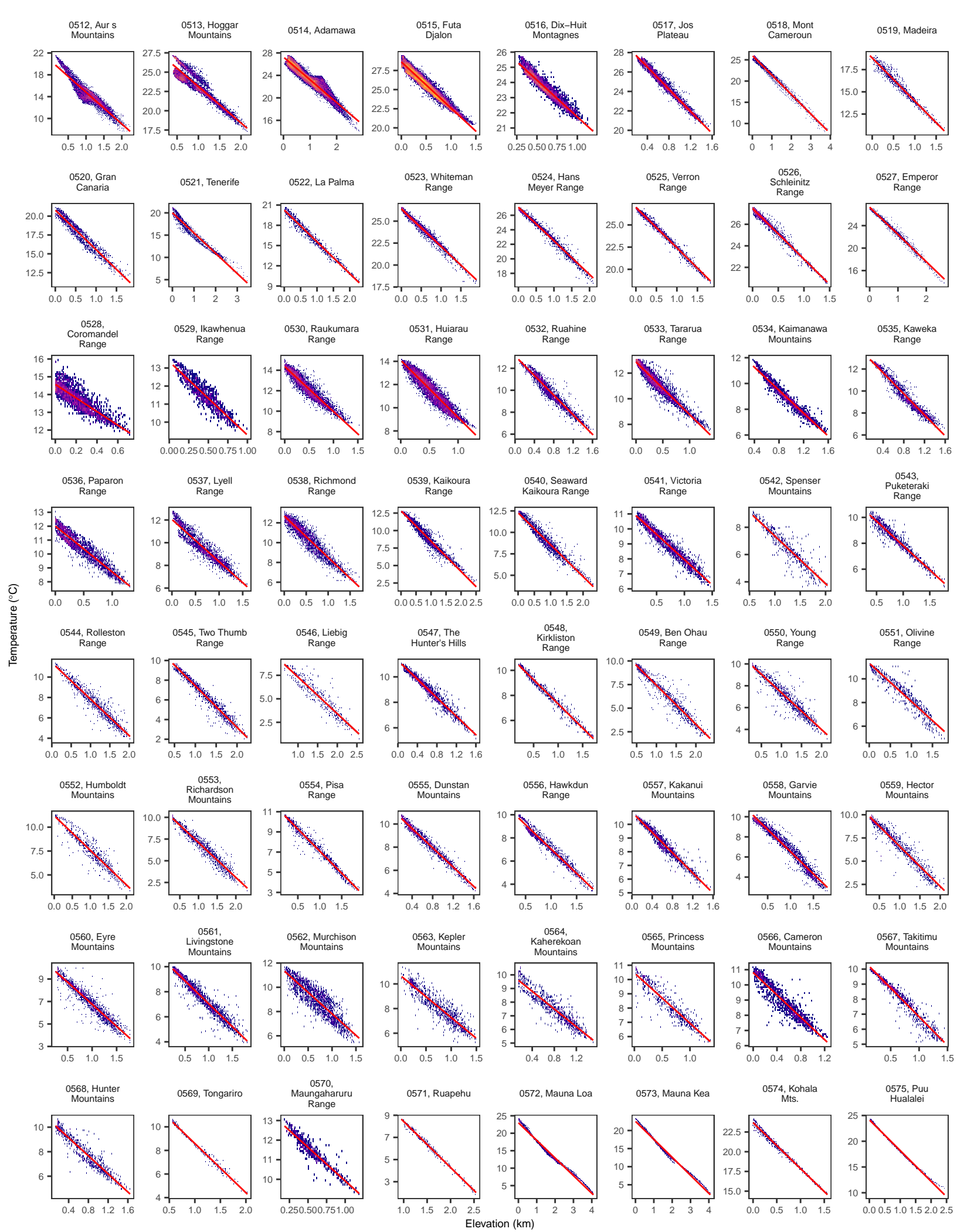

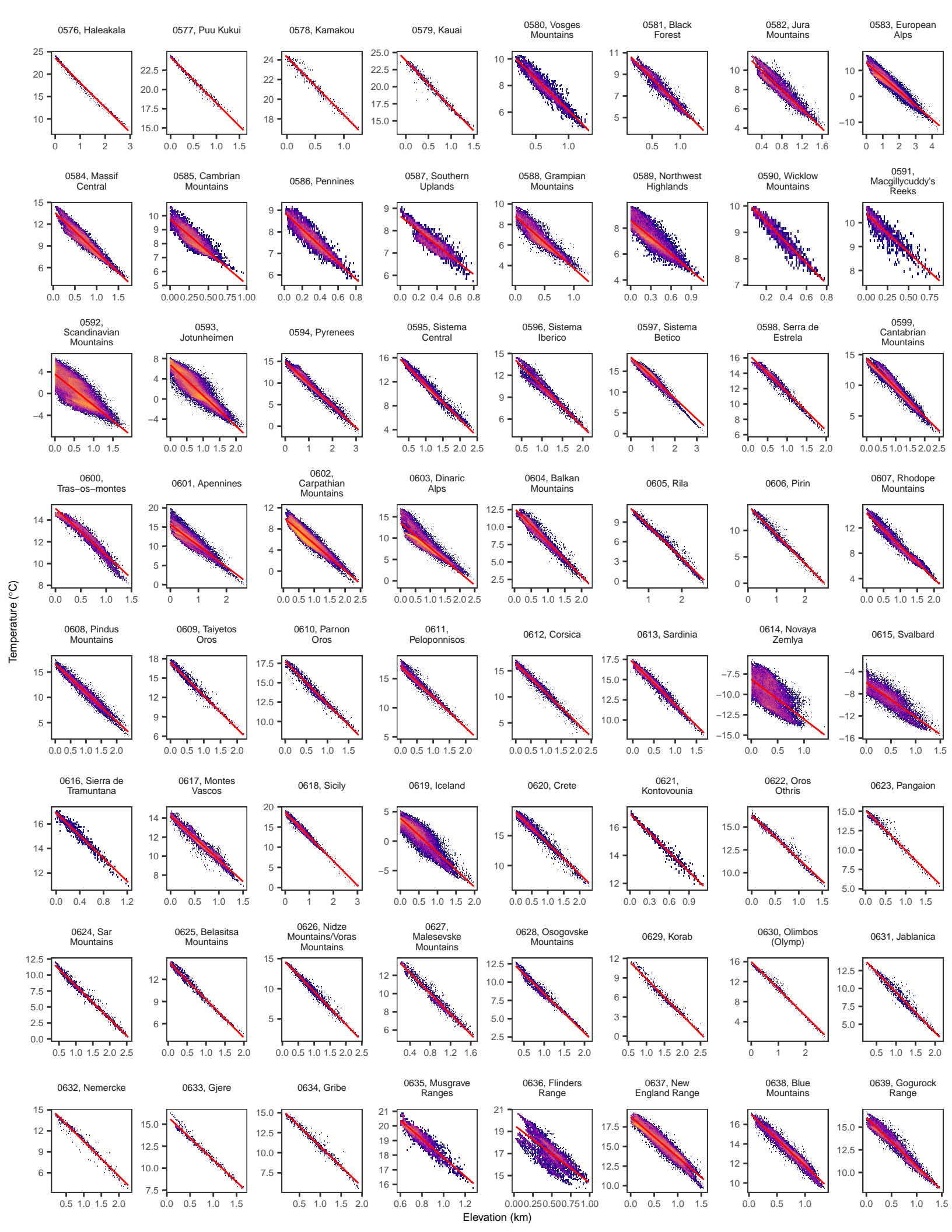

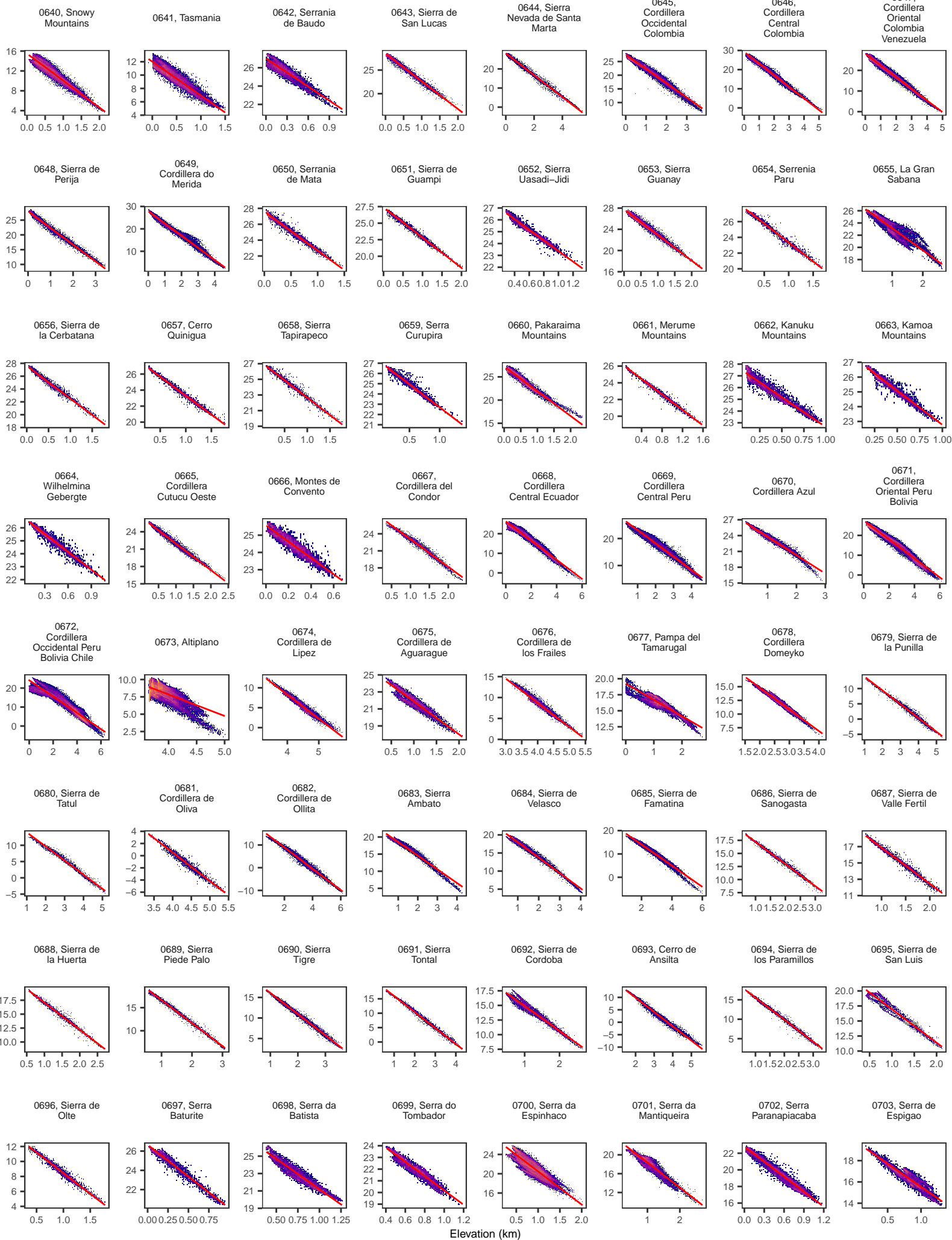

Temperature (°C)

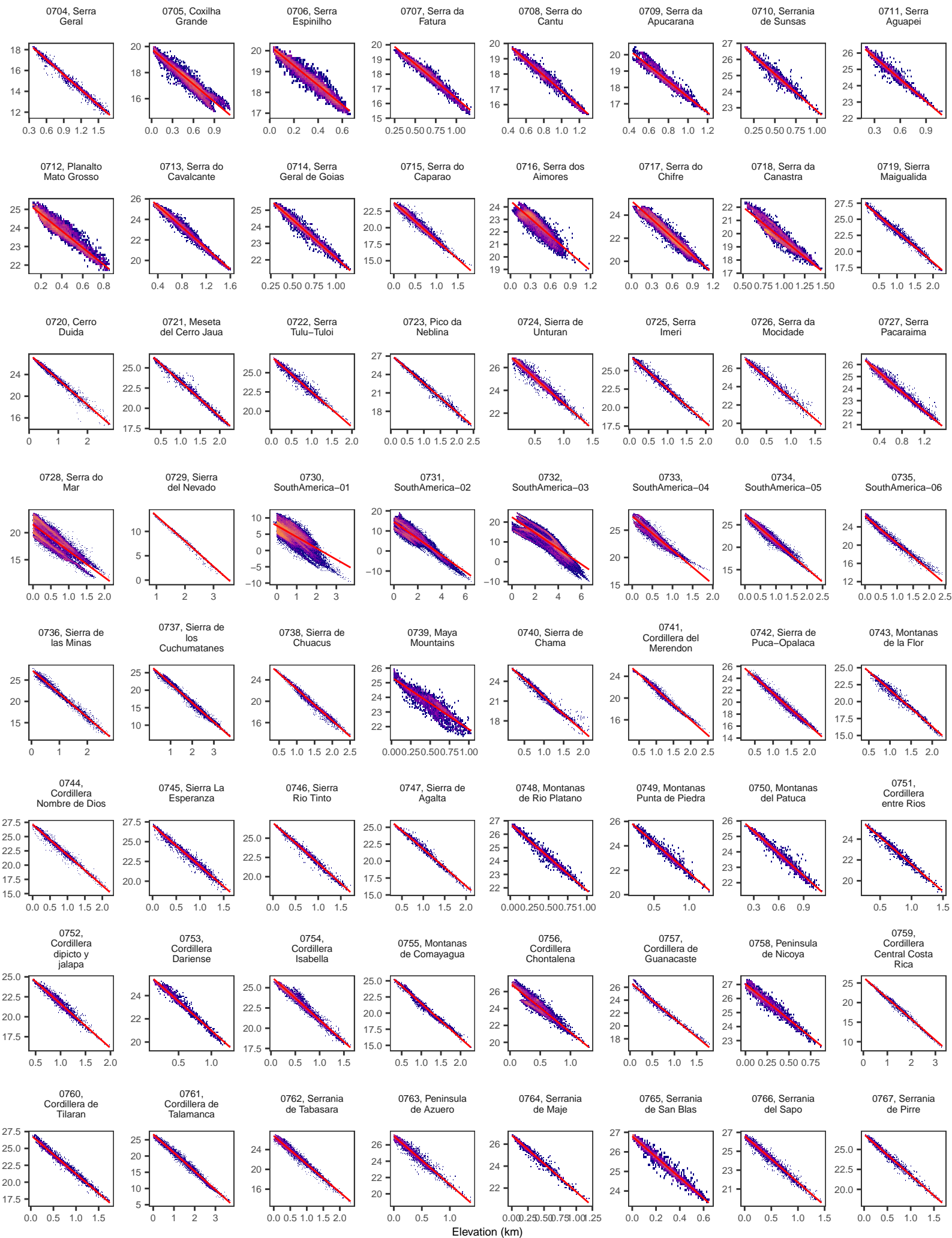

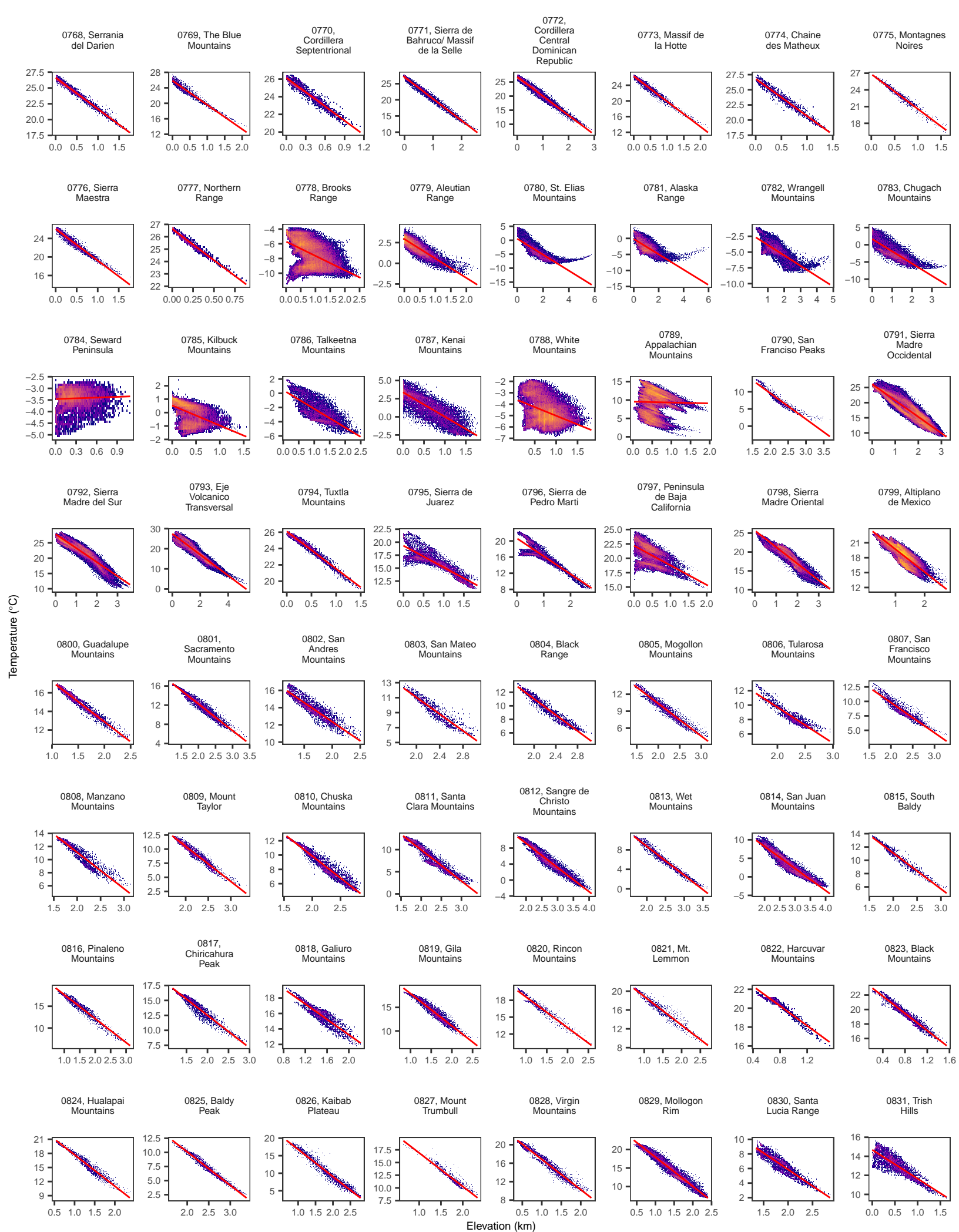

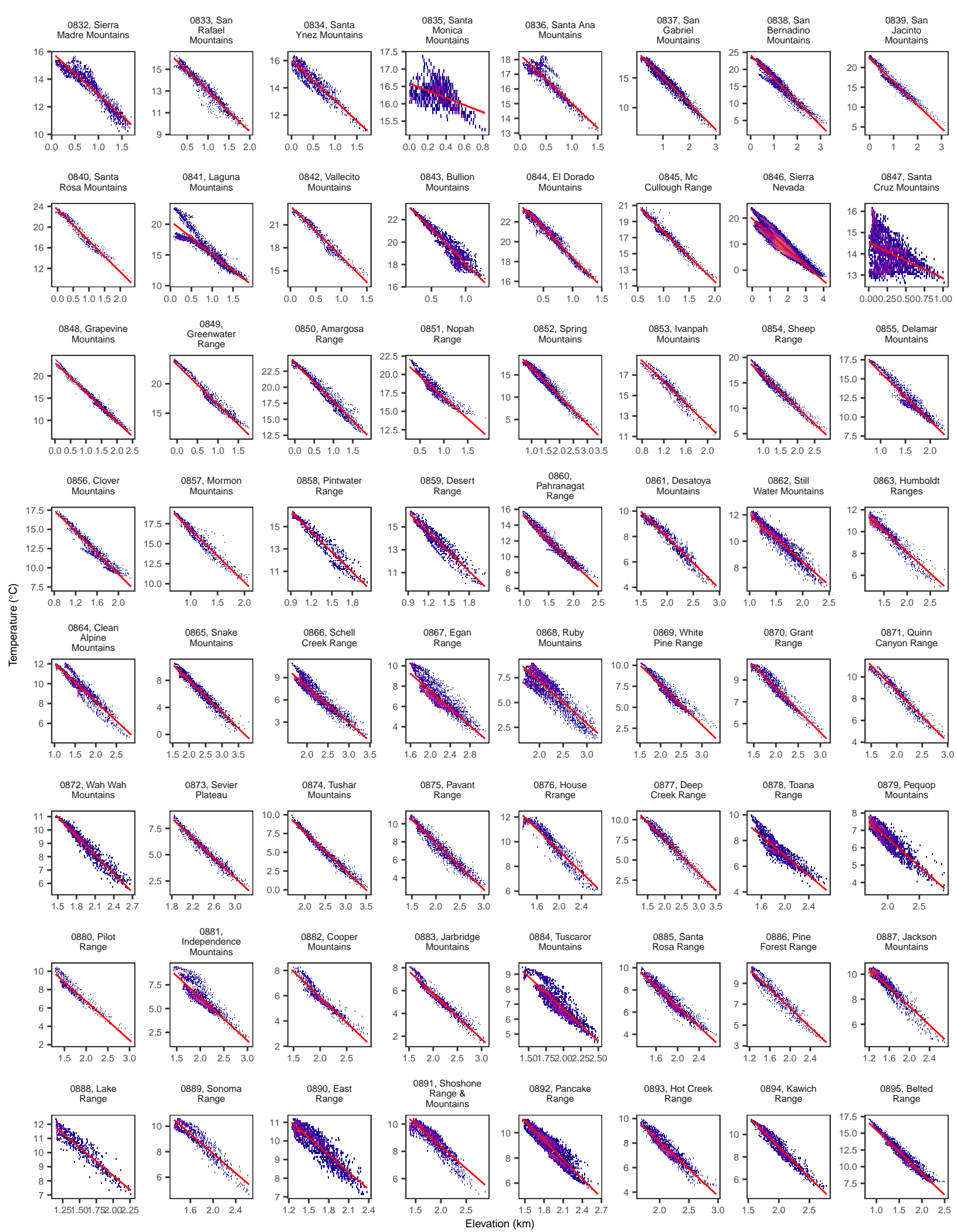

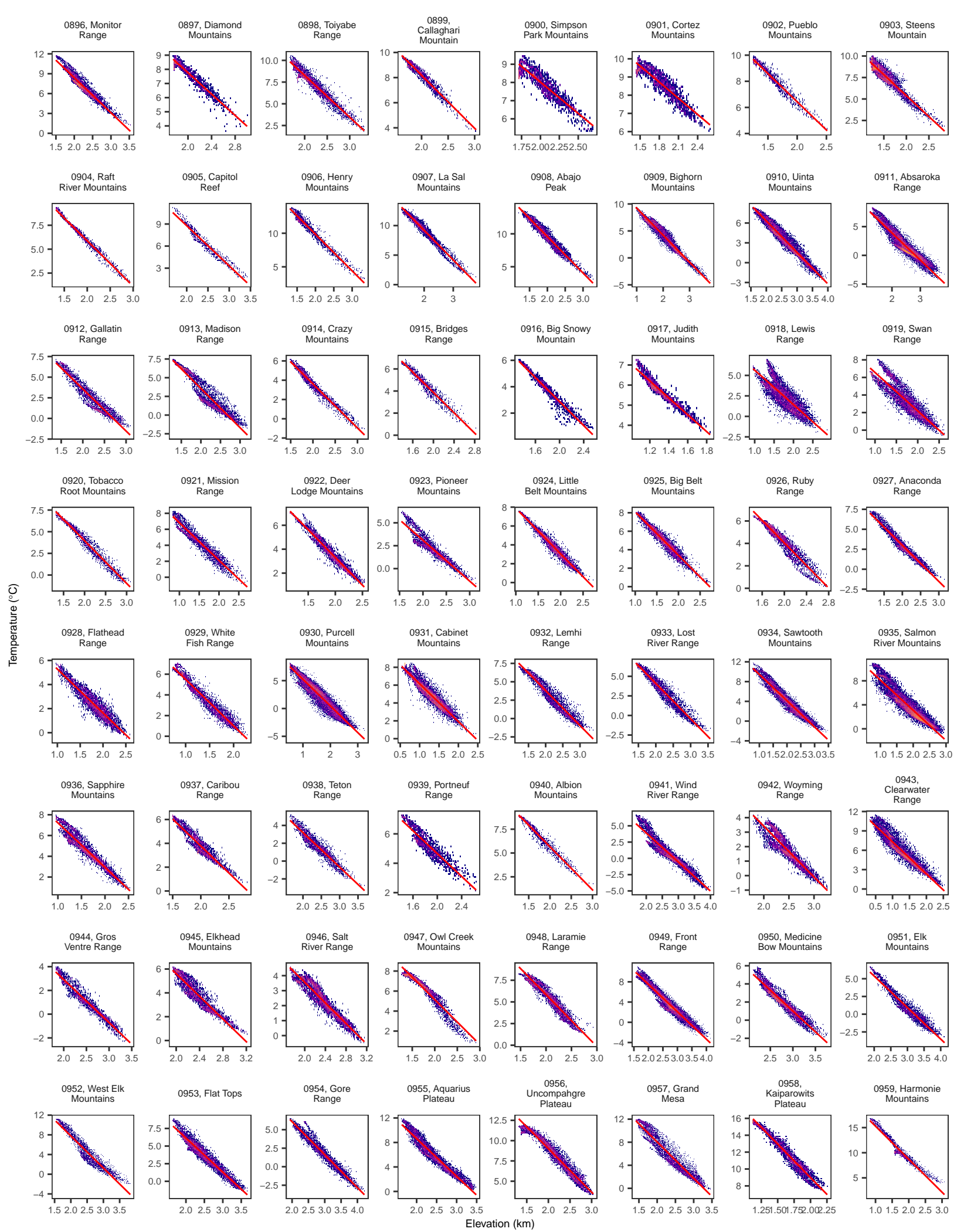

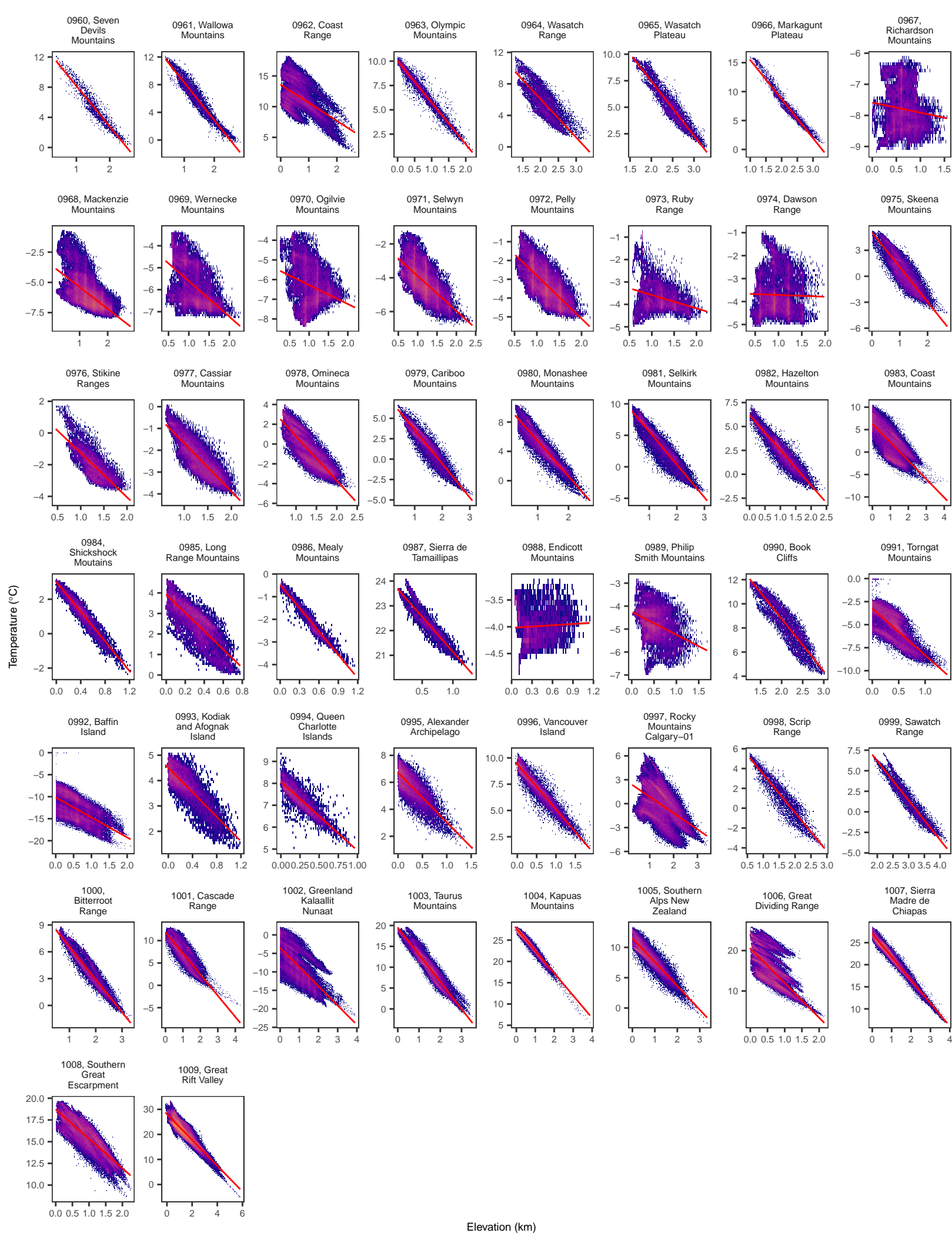

Supplement: Supplementary file 6 — Supplementary Data 2 [file 41467_2020_15881_MOESM6_ESM.pdf]
