# Supplementary material for: Topography and human pressure in mountain ranges alter expected species responses to climate change
Source: Nat Commun. 2020 Apr 24;11:1974. doi: 10.1038/s41467-020-15881-x (PMC7181879; doi:10.1038/s41467-020-15881-x)
Supplement: Supplementary file 7 — Supplementary Software 1 [file 41467_2020_15881_MOESM7_ESM.zip › readme.pdf]

README

The included software and materials files include two folders (ÔcodeÕ and ÔdataÕ). The code folder contains two files; the data folder contains four files.

The ÔcodeÕ folder contains two scripts to be run in the software R. This software is freely available for both PCs and Macs and can be downloaded here: https://cran.r-project.org/mirrors.html. Expected download and install time is <5 minutes.

For testing, we used R version 3.6.1 with a Mac computer with 16 GB RAM, but the scripts should be executable with much less memory and with previous (and later) versions of R, assuming the necessary packages are available. For each script to run properly, the user will need to ensure that all packages listed in the sections denoted Ôload packagesÕ in the scripts are installed. Example code is supplied for reviewers to do this. Once the packages are installed, the code can run from start to finish. Scripts should be run in the order Ô01_mtn_classificationÕ followed by Ô02_range_shift_R1Õ.

Script Ô01_mtn_classificationÕ performs all the necessary functions to classify mountain topography based on all land area and intact land area only (see Figure 1 in submitted manuscript). The script performs this procedure for one example mountain range, the Himalaya, included in the data file Ôsample_data.csvÕ). The code will produce figures analogous to Fig. 1c and assign the range to an initial classification (as in Fig. 1a) and reclassify the range based on intact land area (as in Fig. 1b). The script outputs the necessary data to run script Ô02_range_shift_R1Õ (also provided in the ÔdataÕ folder). Expected run time for this script is <1 minute.

Script Ô02_range_shift_R1Õ performs all the necessary functions to simulate elevational range shifts for a series of hypothetical montane species with different elevational range sizes. The script will load the output data from the previous script, which is also already provided as Ô01_processed_data.csvÕ, along with two pre-processed files containing adiabatic lapse rates and warning rates for two warming scenarios (RCPs) for all 1,010 mountain ranges. The function in the script calculates the amount of total and intact land area each modeled speciesÕ initially occupies prior to the range shift, and then calculates the amount of total and intact land area the speciesÕ occupies following the range shift, using the mountain range-specific adiabatic lapse rate and warming rates. This process is repeated every 50-m of elevation, starting from the mountainÕs base, and calculations are made separately for both warming scenarios. The mean and standard error change in area (as a percent of the initial range size) for each elevation bin is also calculated across all elevational range sizes considered. The code will produce the inset figure in Figure 2 in the submitted manuscript, plotting the change in area for each of the two cases (total and intact), for both warming scenarios. It will also produce the ratio statistic used to color the mountain ranges in Figure 2. Optionally, users can uncomment the last section to plot and explore the results of the different elevational range sizes (not presented in the manuscript). Expected run time for this script is <1 minute.

Reproducing all quantitative results in the manuscript requires running the above scripts over all 1,010 mountain ranges considered in the analysis, and computing summary statistics, including means and standard errors
